# ProQM-resample: improved model quality assessment for membrane proteins by limited conformational sampling

**DOI:** 10.1093/bioinformatics/btu187

**Published:** 2014-04-08

**Authors:** Björn Wallner

**Affiliations:** ^1^Department of Physics, Chemistry and Biology, Linköping University, SE-581 83 Linköping and ^2^Swedish e-Science Research Center, Stockholm, Sweden

## Abstract

**Summary:** Model Quality Assessment Programs (MQAPs) are used to predict the quality of modeled protein structures. These usually use two approaches: methods using consensus of many alternative models and methods requiring only a single model to do its prediction. The consensus methods are useful to improve overall accuracy; however, they frequently fail to pick out the best possible model and cannot be used to generate and score new structures. Single-model methods, on the other hand, do not have these inherent shortcomings and can be used to both sample new structures and improve existing consensus methods. Here, we present *ProQM-resample*, a membrane protein-specific single-model MQAP, that couples side-chain resampling with MQAP rescoring by ProQM to improve model selection. The side-chain resampling is able to improve side-chain packing for 96% of all models, and improve model selection by 24% as measured by the sum of the Z-score for the first-ranked model (from 25.0 to 31.1), even better than the state-of-the-art consensus method Pcons. The improved model selection can be attributed to the improved side-chain quality, which enables the MQAP to rescue good backbone models with poor side-chain packing.

**Availability and implementation:** http://proqm.wallnerlab.org/download/.

**Contact:** bjornw@ifm.liu.se

**Supplementary information:** Supplementary data are available at *Bioinformatics* online.

## 1 INTRODUCTION

Protein structure modeling represents a fundamental challenge in structural bioinformatics and is crucial for a detailed understanding of the structure and biological function of molecules. It can be used to guide and explain experiments, as well as for prediction of proteins whose structure, in particular for membrane proteins, for the most part is unknown (∼0.5% membrane protein in the Protein Data Bank ∼25% in most genomes). A common technique in structure modeling is to generate many alternative models and use a Model Quality Assessment Program (MQAP) to select the best model. Alternatively, an MQAP can also be used to assess the absolute quality of a single model, i.e. a measure that is related to similarity to true native structure (Wallner and Elofsson, 2003; Wang *et al.*, 2009).

ProQM (Ray *et al.*, 2010) is an MQAP that uses a support vector machine to predict the quality of a membrane protein model by combining structural and sequence-based features calculated from the model. In its original implementation, external programs were used to calculate features, e.g. PSI-BLAST (Altschul *et al.*, 1997), PSIPRED (McGuffin *et al.*, 2000), Naccess (Hubbard and Thornton, 1993), Stride (Frishman and Argos, 1995), ProQres (Wallner and Elofsson, 2006), Zpred (Granseth *et al.*, 2006), Topcons (Bernsel *et al.*, 2009), MPRAP (Illergård *et al.*, 2010) and SVM-light (Joachims, 2002). These dependencies made it difficult to distribute the program, run large batches and use it in conformational sampling. Therefore, ProQM has only been available as a webserver for small-scale use. To overcome these issues, ProQM was incorporated as scoring function in the Rosetta modeling framework. This gives in one hand full access to the modeling machinery within Rosetta and allows for easy integration with any Rosetta protocol. In particular, ProQM-resample uses the repack protocol to sample side-chain conformations followed by rescoring using ProQM to improve model selection.

## 2 METHOD DEVELOPMENT

ProQM (Ray *et al.*, 2010) was implemented as a scoring function in Rosetta (Das and Baker, 2008). ProQM uses two sets of features, one that only depends on the model sequence and one that is calculated from the structural model. The sequence-based features only need to be calculated once for a given sequence and are used as input to Rosetta. While all structural features such as atom–atom contacts, residue–residue contacts, surface areas and secondary structures, as well as the SVM prediction are calculated by Rosetta during scoring, there is still a dependency on external programs for the sequence-based features. The programs and the scripts to prepare input files are provided on the download page. There is also a server available at: http://ProQSeq.wallnerlab.org/.

For the structural-based features, we adapted an already existing implementation of Naccess (Hubbard and Thornton, 1993) and DSSP (Kabsch and Sander, 1983) to calculate exposed residue surface area and for assigning secondary structure, respectively. The atom–atom and residue–residue contact matrices, previously calculated by ProQres, were implemented directly in Rosetta as well as the functionality to read and predict SVM models. To account for changes in implementation details, the SVM model weights were retrained using the original 5-fold cross-validated ProQM training set (Ray *et al.,* 2010).

### 2.1 ProQM resampling protocol

**Fig. 1.**
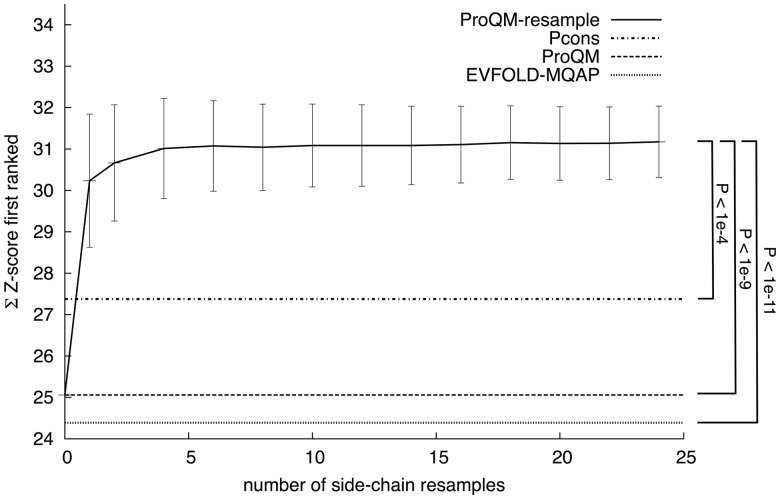
Overall target selection for ProQM-resample measured by the sum of the Z-score for first-ranked models against the number of generated resampled models; Pcons, ProQM and EVFOLD-MQAP are included as reference; error bars correspond to SD resulting from 100 replicas; these were also used to calculate the *P*-values

An advantage of implementing ProQM as a scoring function in the Rosetta modeling framework is that it enables conformational sampling using the MQAP as part of the scoring function. However, because an MQAP should measure the quality of any input model it cannot make large change to the model and claim it is assessing the quality of the input model. Therefore, we decided to sample only the side-chain rotamers while keeping the backbone fixed, effectively keeping the quality measures based on Cα coordinates such as TMscore (Zhang and Skolnick, 2004) constant. This was achieved by rebuilding side-chains with a backbone-dependent rotamer library implemented in the *repack* protocol using the *score_membrane* scoring function (Barth *et al.*, 2007), followed by rescore using ProQM. Based on Figure 1 sampling and rescoring, 10 different model decoys for each initial model seem to be a good choice.

### 2.2 Benchmark

ProQM-resample was benchmarked on the independent EVfold_membrane data set (Hopf *et al.*, 2012) consisting of 15 340 models for 25 targets generated with CNS (Brunger *et al.*, 1998) using distance constraints from evolutionary couplings extracted from large multiple sequence alignments (the set is actually larger, but only 25 targets had a known structure). Results were compared with Pcons (Larsson *et al.*, 2009), a state-of-the-art consensus method, and EVFOLD-MQAP (Hopf *et al.*, 2012), a predicted ranking based on satisfaction of unused constraints, predicted secondary structure and predicted lipid exposure agreement. In the cases where the EVfold_membrane set contained homologous proteins (BLAST E < 0.01) to the original ProQM training set, ProQM was retrained with non-homologous proteins. Model selection accuracy was measured by Z-scores calculated from TMscore.

## 3 RESULTS

First, the ProQM implementation in Rosetta was compared with the original ProQM-webserver version. They should be similar, but because of different implementations of Naccess and DSSP versus STRIDE, and the fixing of some minor bugs, the results will not be identical. Still, there is a clear correlation, *R* = 0.98, between ProQM-Rosetta and ProQM-webserver and the prediction performance is also maintained, *R* = 0.62 to true answer (data not shown).

The benchmark on the EVfold_membrane set showed that model selection is significantly improved by resampling the side-chains (Fig 1). Already without any sampling ProQM selects slightly better models than EVFOLD-MQAP (*Z* = 25.0 versus 24.4). Side-chain sampling increases the performance significantly to *Z* = 31.1 and levels out at around 10 resamples per model, surpassing even the state-of-the-art consensus Pcons. This demonstrates the usefulness of single-model MQAPs in model selection. It also highlights the need to include side-chain sampling into the MQAP procedure to avoid losing good backbone models suffering from poor side-chain packing. A possible reason for the improved selection is the fact that almost all models (96%) improved the side-chain packing after resampling (Supplementary Fig. S1). Before resampling, the side-chain quality is roughly the same for all targets (Table 1). But after resampling the side-chain improvement is larger for the set of targets that also show backbone improvement after resampling compared with targets that show no improvement in backbone, indicating that improved side-chains help the MQAP to select better backbone models.

**Table 1.** Side-chain quality before and after resampling

Set	Before resampling (%)	After resampling (%)	Number of targets	Number of models
All	13.5 ± 0.1	20.5 ± 0.2	25	15 340
No improvement	13.3 ± 0.2	19.0 ± 0.3	11	6538
Improvement	13.6 ± 0.2	22.0 ± 0.3	8	4377

*Note*: Side-chain quality measured by fraction chi1 and chi2 within 40 from correct. Error estimates represent 99.999% confidence intervals. The sets correspond to all models, models from targets without and with backbone improvement. See Supplementary Information for exact definition of the sets.

*Funding*: Swedish Research Council (Dnr 2012-5270) and Carl Tryggers Stiftelse (Dnr 12:516).

*Conflict of Interest*: none declared.

## Supplementary Material

Supplementary Data
