# Supplementary material for: ProQM-resample: improved model quality assessment for membrane proteins by limited conformational sampling
Source: Bioinformatics. 2014 Apr 8;30(15):2221–3. doi: 10.1093/bioinformatics/btu187 (PMC4103597; doi:10.1093/bioinformatics/btu187)
Supplement: Supplementary Data [file supp_30_15_2221__index.html]

ProQM-resample: improved model quality assessment for membrane proteins by limited conformational sampling — ProQM-resample: improved model quality assessment for membrane proteins by limited conformational sampling — Supplementary Data 

# ProQM-resample: improved model quality assessment for membrane proteins by limited conformational sampling

## Supplementary Data

files

**Files in this Data Supplement:**

- Supplementary Data - pdf file
