# Supplementary material for: ProQM-resample: improved model quality assessment for membrane proteins by limited conformational sampling
Source: Bioinformatics. 2014 Apr 8;30(15):2221–3. doi: 10.1093/bioinformatics/btu187 (PMC4103597; doi:10.1093/bioinformatics/btu187)
Supplement: Supplementary Data [file supp_btu187_ProQM-resample_SI.pdf]

Supplementary Information

ProQM-resample: Improved Model Quality Assessment for Membrane Proteins by Limited Conformational Sampling

Björn Wallner[1,2*]

[1]Department of Physics, Chemistry and Biology, Linköping University, SE-581 83 Linköping, Sweden. bjornw@ifm.liu.se

[2]Swedish e-Science Research Center

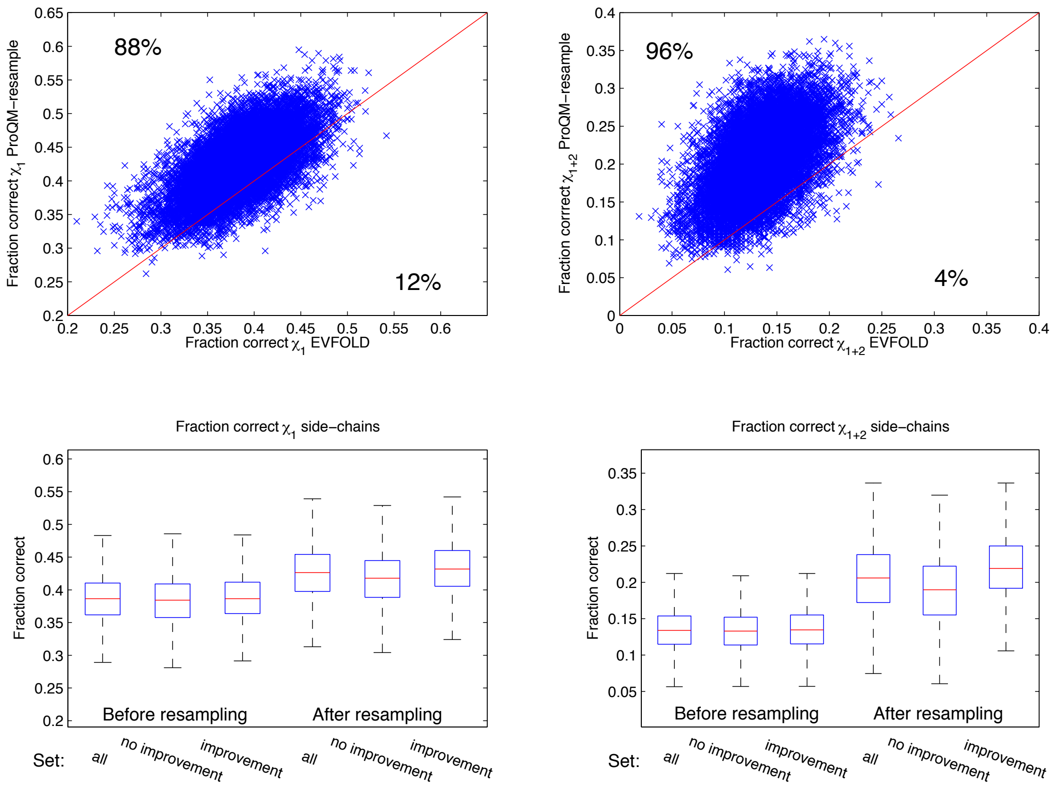

Fig. S1. Side-chain quality before and after resampling, a rotamer is considered correct if it is within 40 degrees of the correct rotamer. A) Fraction correct χ_1 before vs. after resampling; B) Fraction correct χ_{1+2} before vs. after resampling; C) Box-and-whisker plots showing the distribution of fraction correct χ_1 for the complete set (all), the targets showing no improvement in model selection (no improvement), and targets showing improved selection (improvement) before and after resampling, respectively. The median is showed in red, the box represent the first and third quartiles and the whiskers extends to 1.5 of the interquartile range covering 99.3% of the data assuming normal distribution; D) Same as in C) but for χ_{1+2}. The targets included in the *no improvement* and *improvement* sets are defined in Fig S2.

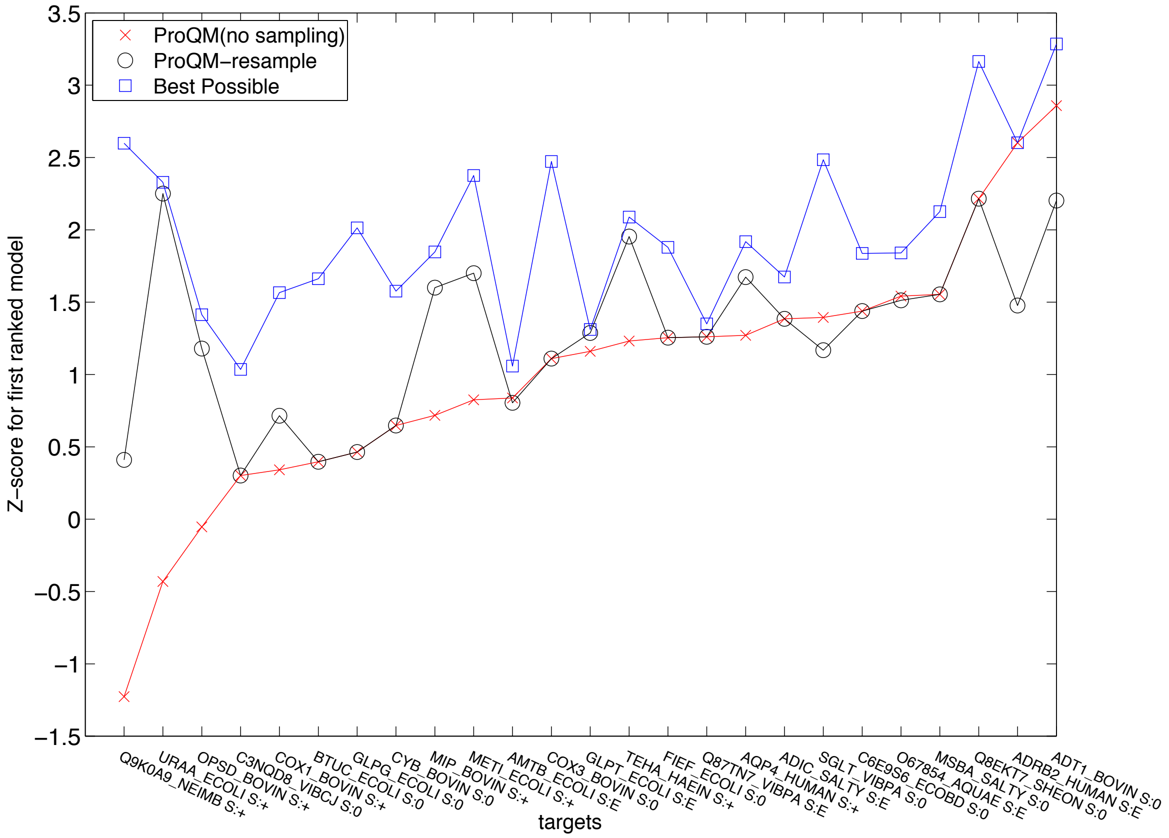

Fig. S2. Target selection per target as measured by the Z-score for the first ranked model for ProQM, ProQM-resampling and the best possible selection, respectively. To analyze the reason for improvement targets were group into three sets, the *excluded* set for which no improvement is possible since the initial choice is already close to optimal, the *improvement* set, and *no improvement* set. Targets with Z_{best}-Z_{ProQM}<0.3 were put in the *excluded* set, marked by S:E after the target name on the x-axis, targets with $Z_{ProQM\text{-}resampling}$-$Z_{ProQM}$=$\Delta Z$>0.3, marked by S:+, are included in the *improvement* set, and targets with and abs(ΔZ)<0.3, marked by S:0, are included in the *no improvement* set.

Fig. S3. Scatter plots of predicted scores for ProQM-resample, ProQM, Pcons and EVFOLD-MQAP vs TMscore for each of the 25 targets. The title of each scatter plot has correlation coefficient, R, the TMscore and the Z score for the top 1 model, and the TMscore and Z score for the best possible model.

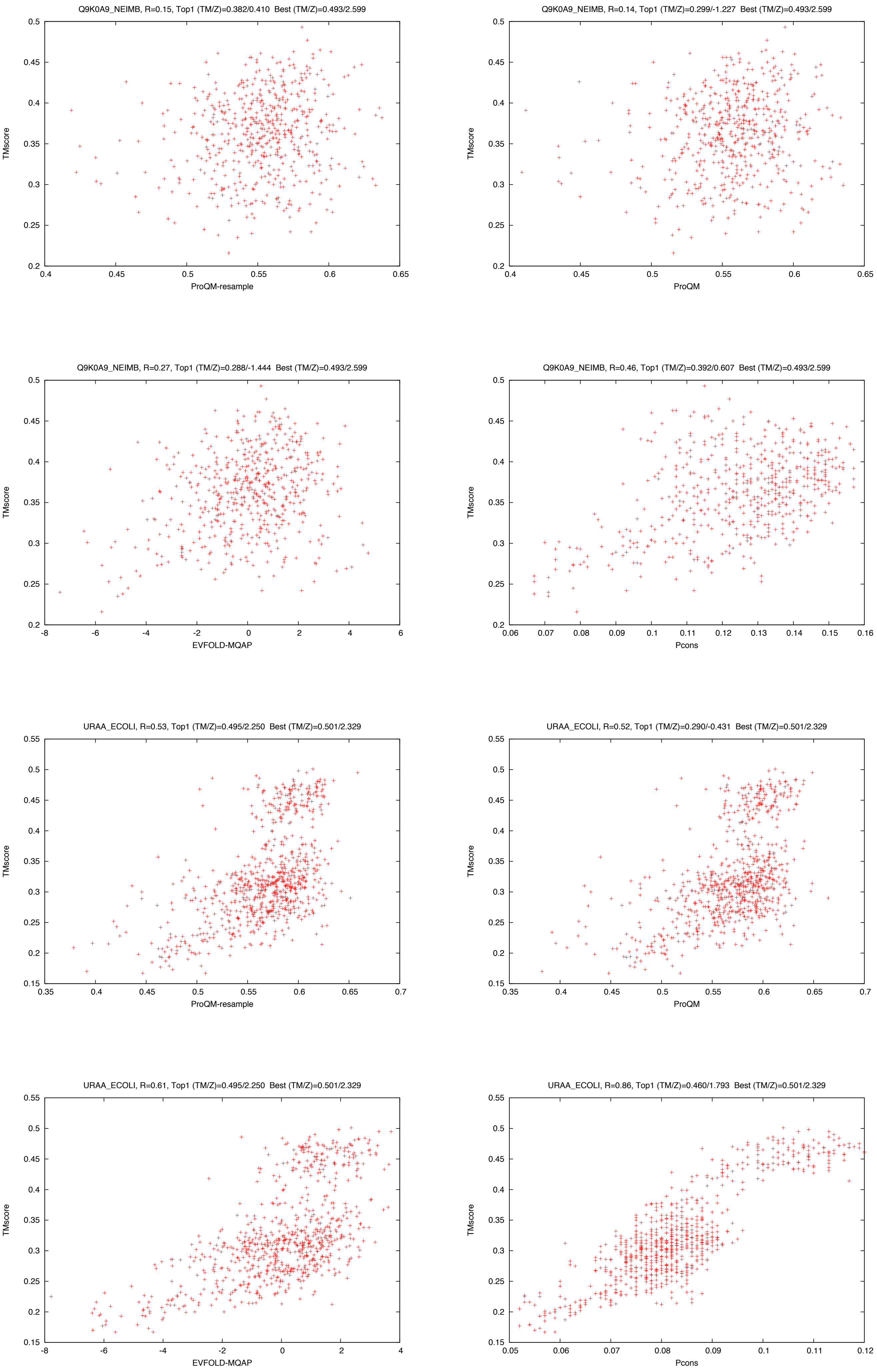
Q9K0A9_NEIMB, R=0.15, Top1 (TM/Z)=0.382/0.410 Best (TM/Z)=0.493/2.599
TMscore
ProQM-resample
Q9K0A9_NEIMB, R=0.14, Top1 (TM/Z)=0.299/-1.227 Best (TM/Z)=0.493/2.599
TMscore
ProQM
Q9K0A9_NEIMB, R=0.27, Top1 (TM/Z)=0.288/-1.444 Best (TM/Z)=0.493/2.599
TMscore
EVFOLD-MQAP
Q9K0A9_NEIMB, R=0.46, Top1 (TM/Z)=0.392/0.607 Best (TM/Z)=0.493/2.599
TMscore
Pcons
URAA_ECOLI, R=0.53, Top1 (TM/Z)=0.495/2.250 Best (TM/Z)=0.501/2.329
TMscore
ProQM-resample
URAA_ECOLI, R=0.52, Top1 (TM/Z)=0.290/-0.431 Best (TM/Z)=0.501/2.329
TMscore
ProQM
URAA_ECOLI, R=0.61, Top1 (TM/Z)=0.495/2.250 Best (TM/Z)=0.501/2.329
TMscore
EVFOLD-MQAP
URAA_ECOLI, R=0.86, Top1 (TM/Z)=0.460/1.793 Best (TM/Z)=0.501/2.329
TMscore
Pcons

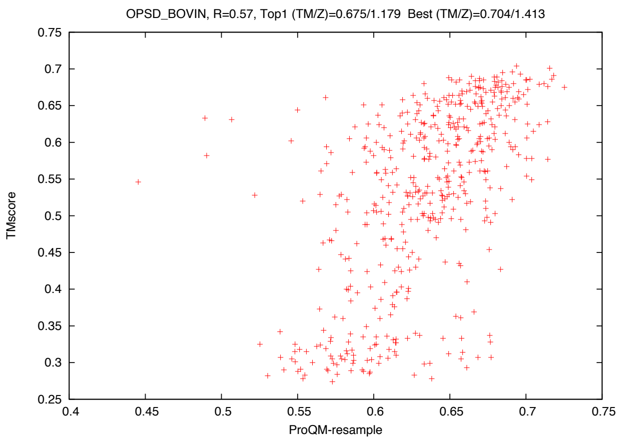
OPSD_BOVIN, R=0.57, Top1 (TM/Z)=0.675/1.179 Best (TM/Z)=0.704/1.413
TMscore
ProQM-resample

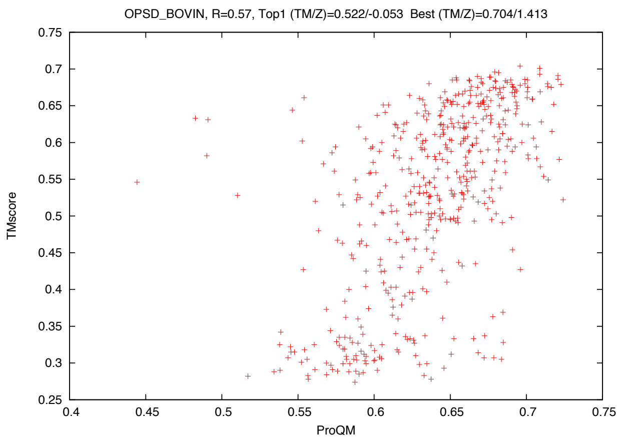
OPSD_BOVIN, R=0.57, Top1 (TM/Z)=0.522/-0.053 Best (TM/Z)=0.704/1.413
TMscore
ProQM

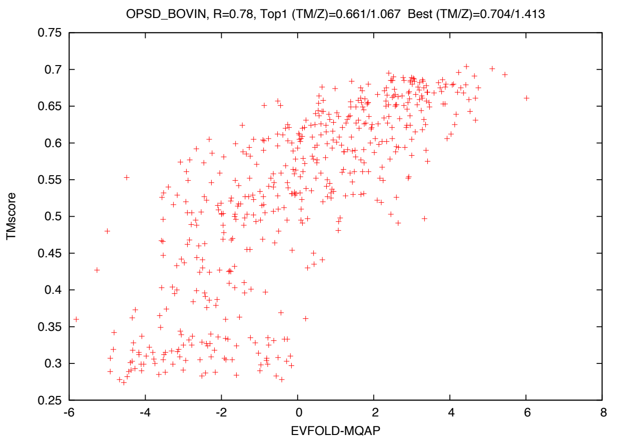
OPSD_BOVIN, R=0.78, Top1 (TM/Z)=0.661/1.067 Best (TM/Z)=0.704/1.413
TMscore
EVFOLD-MQAP

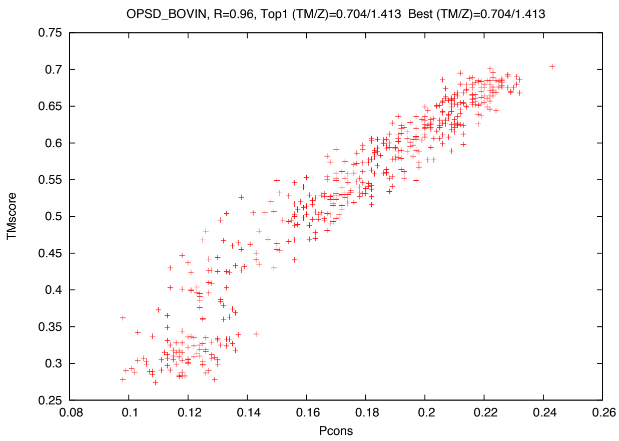
OPSD_BOVIN, R=0.96, Top1 (TM/Z)=0.704/1.413 Best (TM/Z)=0.704/1.413
TMscore
Pcons

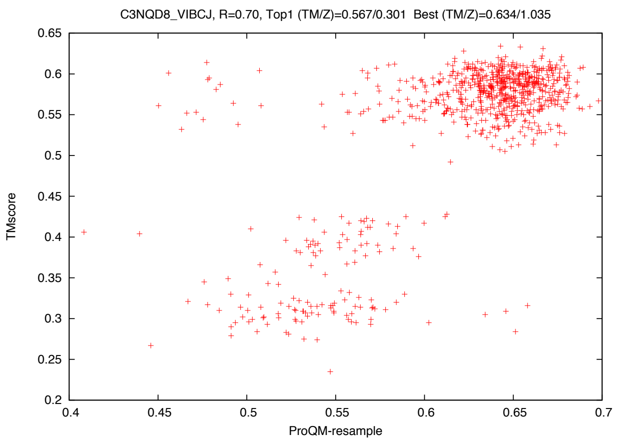
C3NQD8_VIBCJ, R=0.70, Top1 (TM/Z)=0.567/0.301 Best (TM/Z)=0.634/1.035
TMscore
ProQM-resample

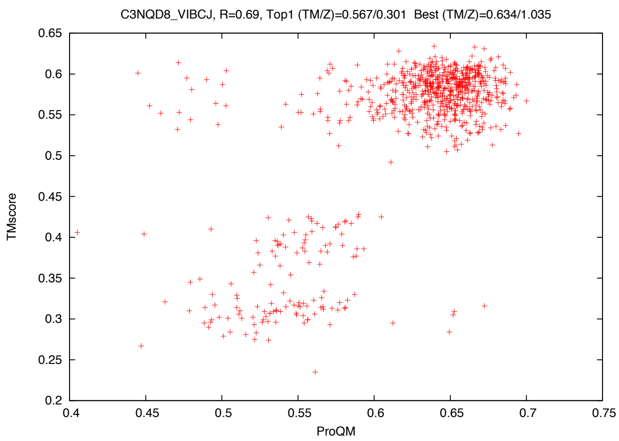
C3NQD8_VIBCJ, R=0.69, Top1 (TM/Z)=0.567/0.301 Best (TM/Z)=0.634/1.035
TMscore
ProQM

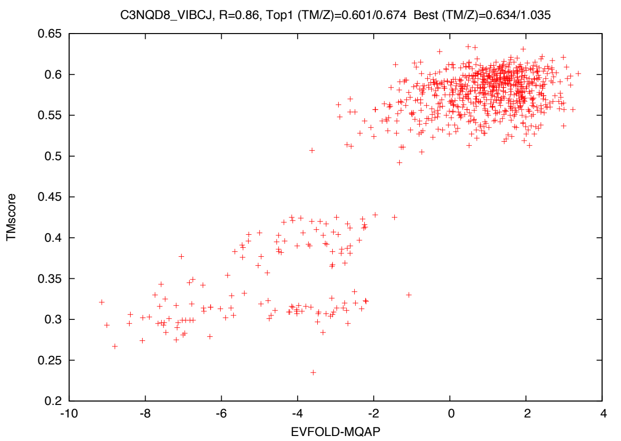
C3NQD8_VIBCJ, R=0.86, Top1 (TM/Z)=0.601/0.674 Best (TM/Z)=0.634/1.035
TMscore
EVFOLD-MQAP

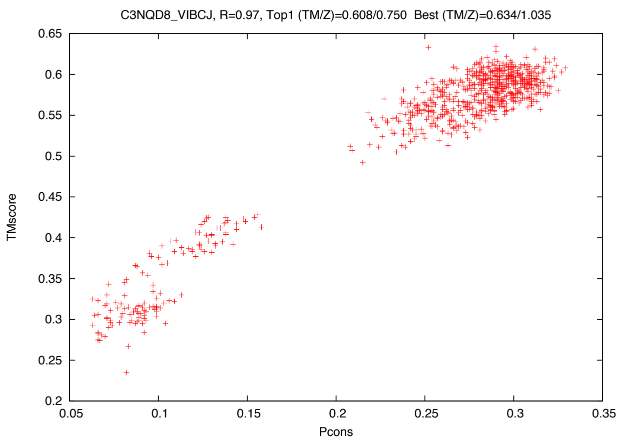
C3NQD8_VIBCJ, R=0.97, Top1 (TM/Z)=0.608/0.750 Best (TM/Z)=0.634/1.035
TMscore
Pcons

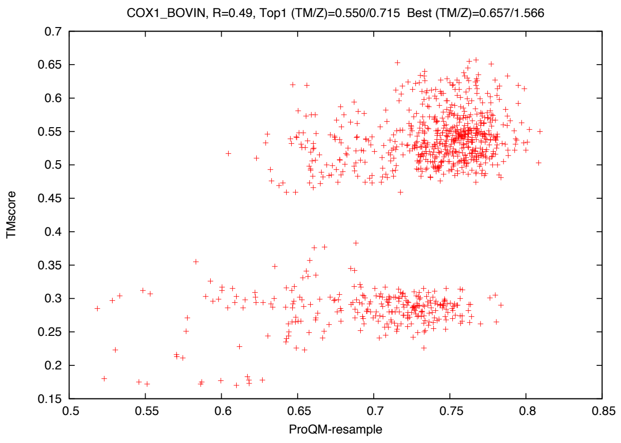
COX1_BOVIN, R=0.49, Top1 (TM/Z)=0.550/0.715 Best (TM/Z)=0.657/1.566
TMscore
ProQM-resample

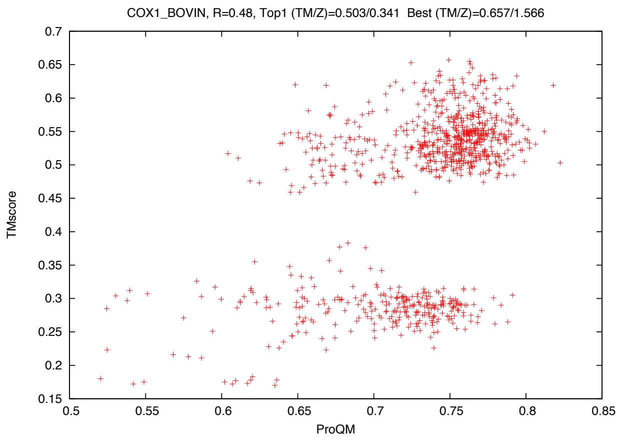
COX1_BOVIN, R=0.48, Top1 (TM/Z)=0.503/0.341 Best (TM/Z)=0.657/1.566
TMscore
ProQM

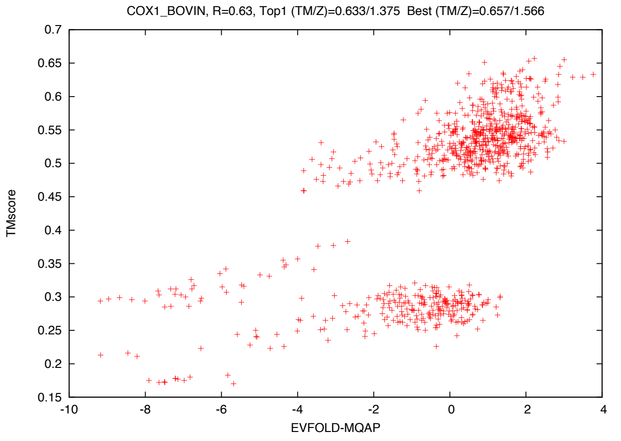
COX1_BOVIN, R=0.63, Top1 (TM/Z)=0.633/1.375 Best (TM/Z)=0.657/1.566
TMscore
EVFOLD-MQAP

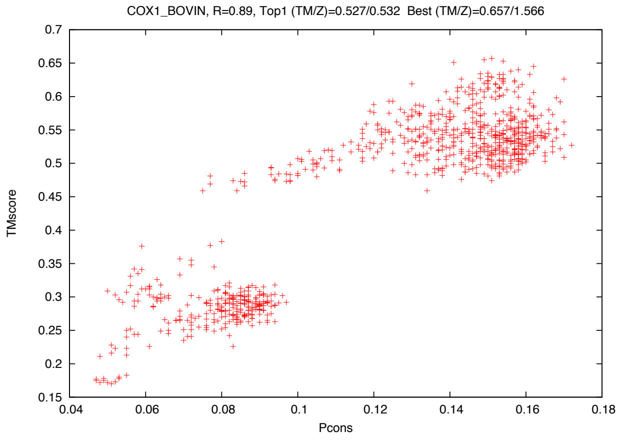
COX1_BOVIN, R=0.89, Top1 (TM/Z)=0.527/0.532 Best (TM/Z)=0.657/1.566
TMscore
Pcons

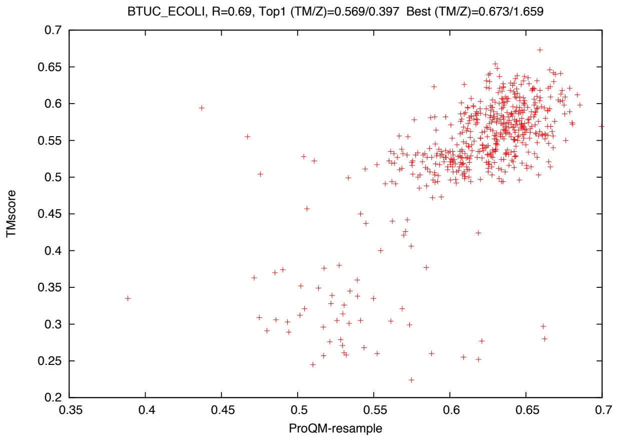
BTUC_ECOLI, R=0.69, Top1 (TM/Z)=0.569/0.397 Best (TM/Z)=0.673/1.659
TMscore
ProQM-resample

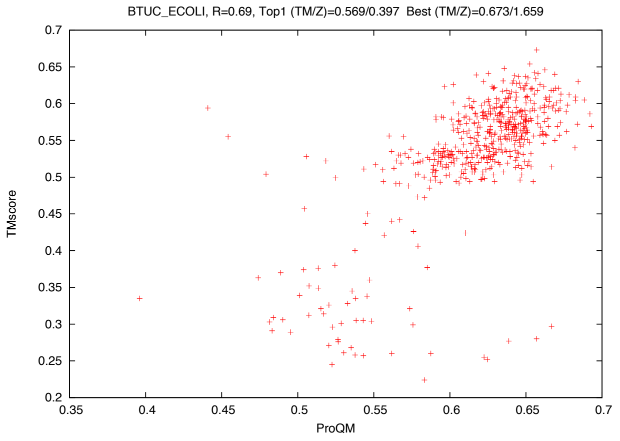
BTUC_ECOLI, R=0.69, Top1 (TM/Z)=0.569/0.397 Best (TM/Z)=0.673/1.659
TMscore
ProQM

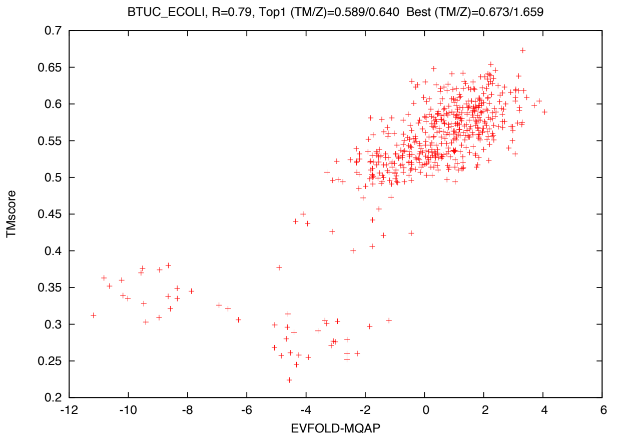
BTUC_ECOLI, R=0.79, Top1 (TM/Z)=0.589/0.640 Best (TM/Z)=0.673/1.659
TMscore
EVFOLD-MQAP

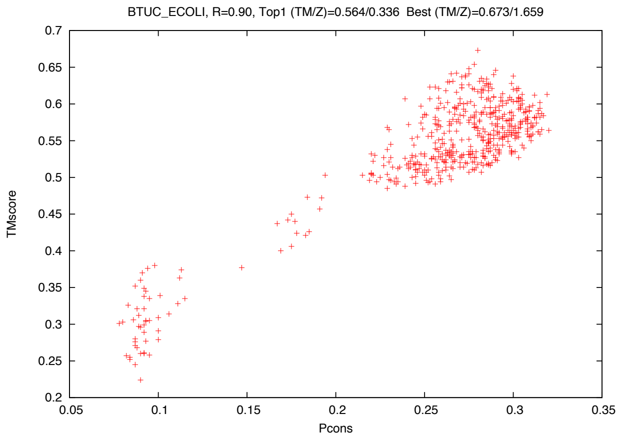
BTUC_ECOLI, R=0.90, Top1 (TM/Z)=0.564/0.336 Best (TM/Z)=0.673/1.659
TMscore
Pcons

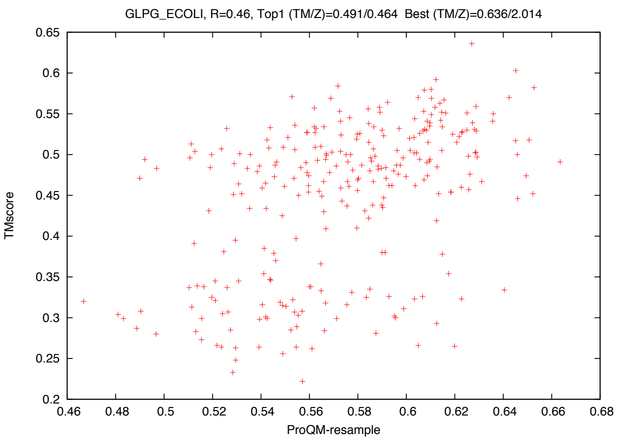
GLPG_ECOLI, R=0.46, Top1 (TM/Z)=0.491/0.464 Best (TM/Z)=0.636/2.014
TMscore
ProQM-resample

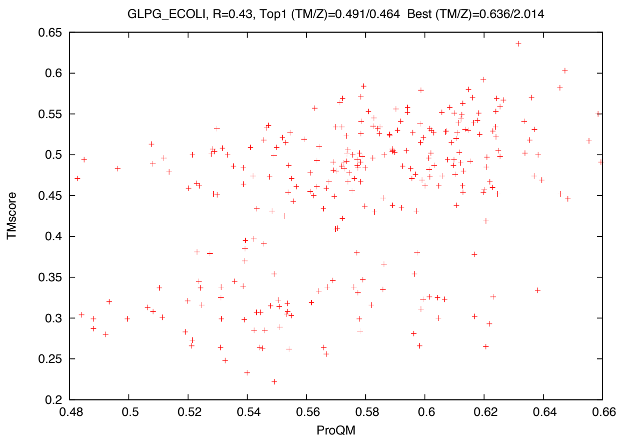
GLPG_ECOLI, R=0.43, Top1 (TM/Z)=0.491/0.464 Best (TM/Z)=0.636/2.014
TMscore
ProQM

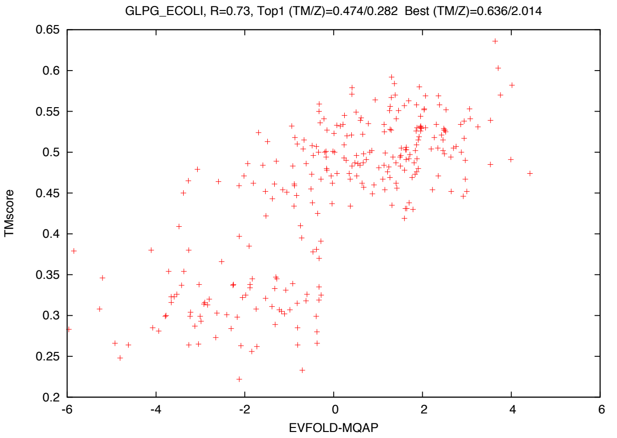
GLPG_ECOLI, R=0.73, Top1 (TM/Z)=0.474/0.282 Best (TM/Z)=0.636/2.014
TMscore
EVFOLD-MQAP

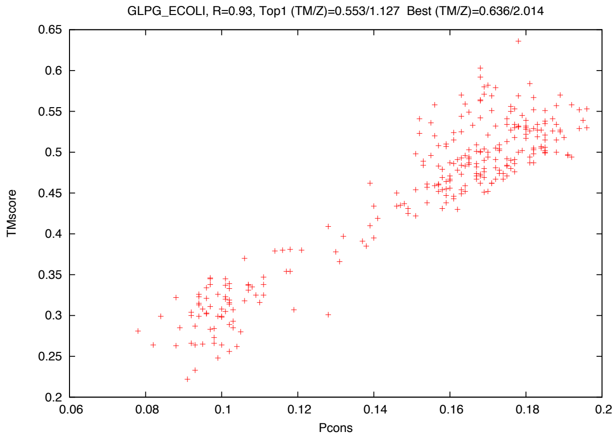
GLPG_ECOLI, R=0.93, Top1 (TM/Z)=0.553/1.127 Best (TM/Z)=0.636/2.014
TMscore
Pcons

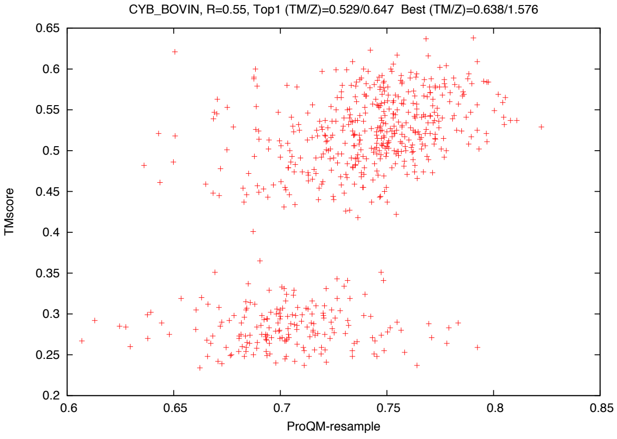
CYB_BOVIN, R=0.55, Top1 (TM/Z)=0.529/0.647 Best (TM/Z)=0.638/1.576
TMscore
ProQM-resample

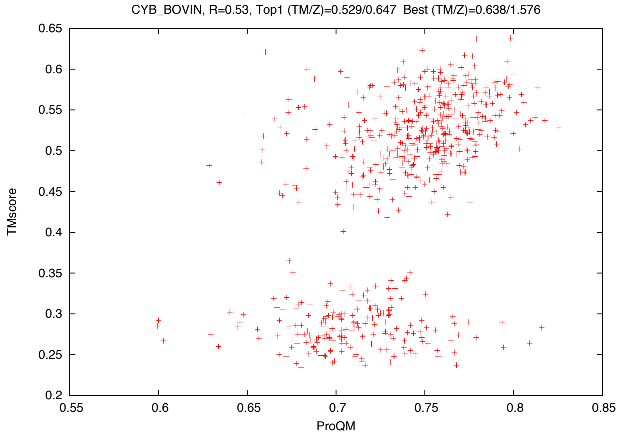
CYB_BOVIN, R=0.53, Top1 (TM/Z)=0.529/0.647 Best (TM/Z)=0.638/1.576
TMscore
ProQM

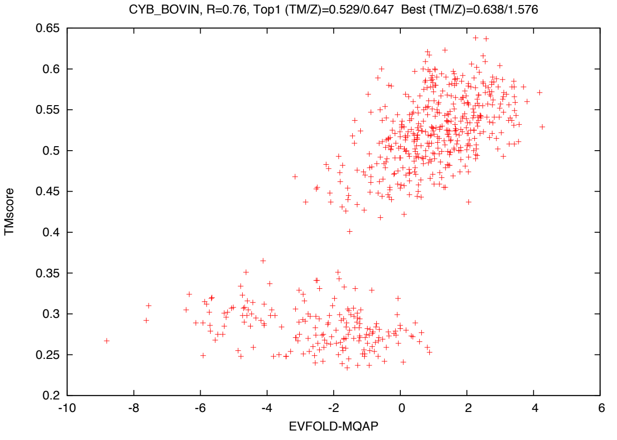
CYB_BOVIN, R=0.76, Top1 (TM/Z)=0.529/0.647 Best (TM/Z)=0.638/1.576
TMscore
EVFOLD-MQAP

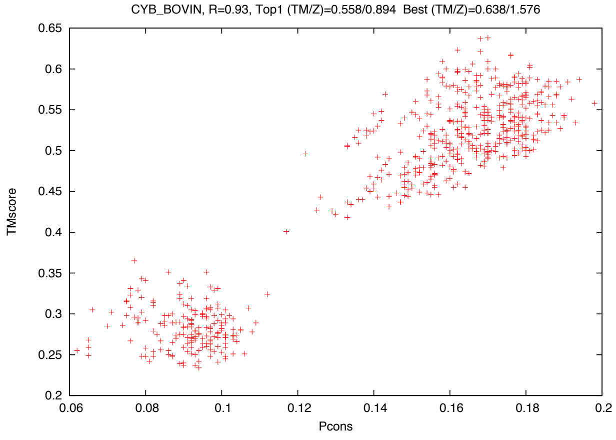
CYB_BOVIN, R=0.93, Top1 (TM/Z)=0.558/0.894 Best (TM/Z)=0.638/1.576
TMscore
Pcons

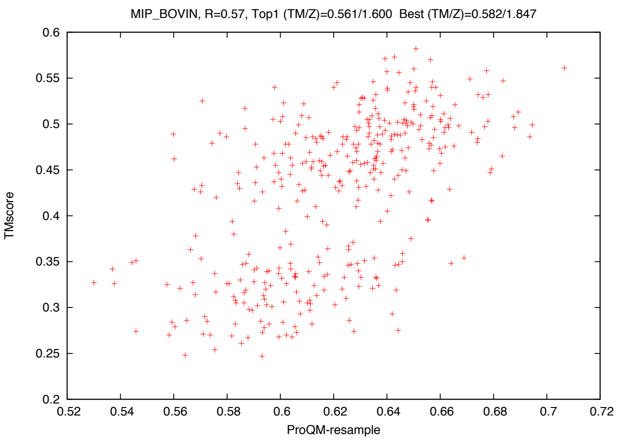
MIP_BOVIN, R=0.57, Top1 (TM/Z)=0.561/1.600 Best (TM/Z)=0.582/1.847
TMscore
ProQM-resample

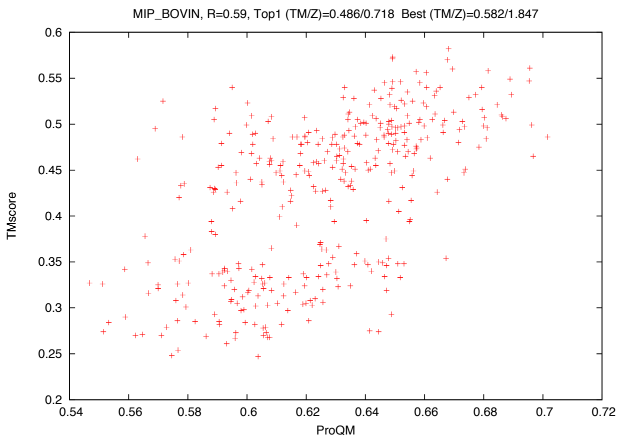
MIP_BOVIN, R=0.59, Top1 (TM/Z)=0.486/0.718 Best (TM/Z)=0.582/1.847
TMscore
ProQM

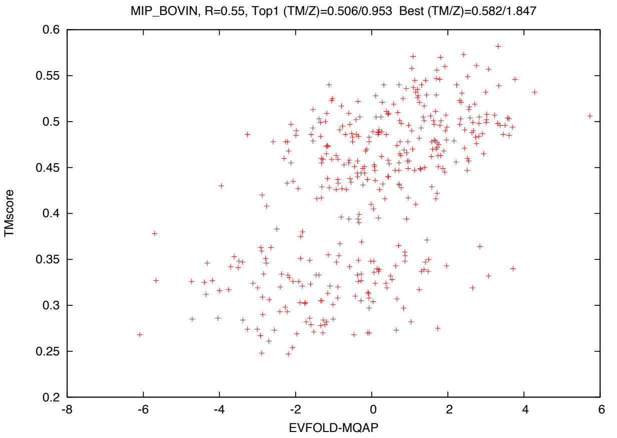
MIP_BOVIN, R=0.55, Top1 (TM/Z)=0.506/0.953 Best (TM/Z)=0.582/1.847
TMscore
EVFOLD-MQAP

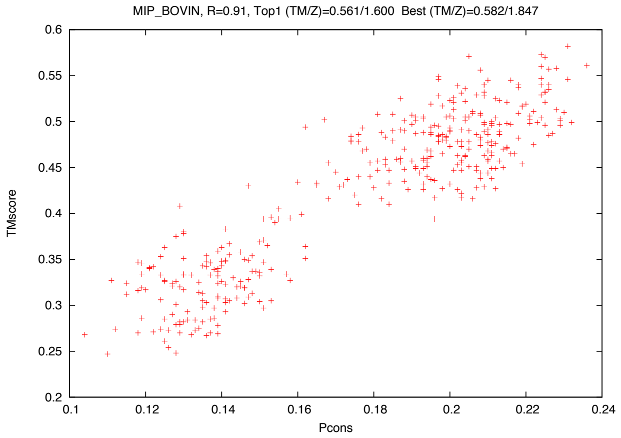
MIP_BOVIN, R=0.91, Top1 (TM/Z)=0.561/1.600 Best (TM/Z)=0.582/1.847
TMscore
Pcons

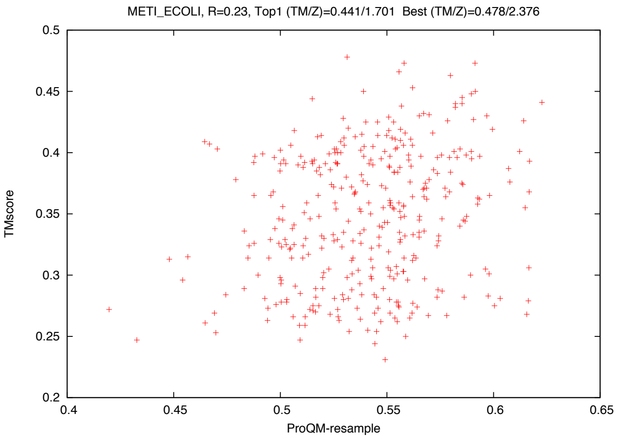
METI_ECOLI, R=0.23, Top1 (TM/Z)=0.441/1.701 Best (TM/Z)=0.478/2.376
TMscore
ProQM-resample

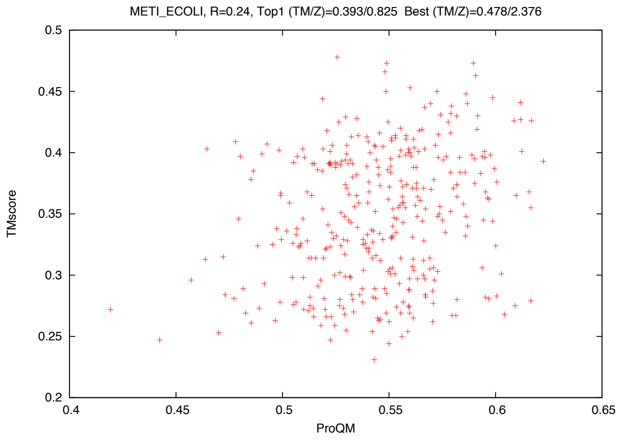
METI_ECOLI, R=0.24, Top1 (TM/Z)=0.393/0.825 Best (TM/Z)=0.478/2.376
TMscore
ProQM

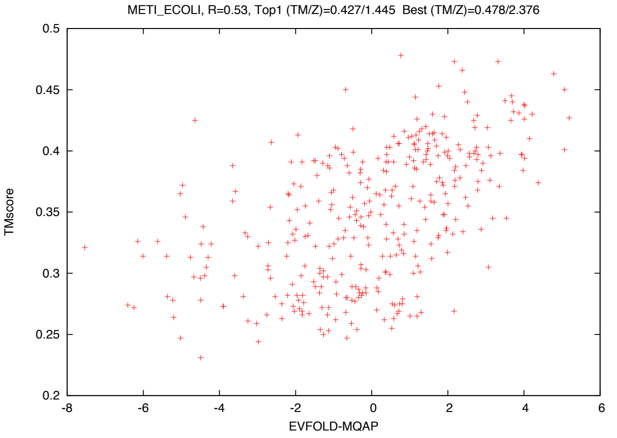
METI_ECOLI, R=0.53, Top1 (TM/Z)=0.427/1.445 Best (TM/Z)=0.478/2.376
TMscore
EVFOLD-MQAP

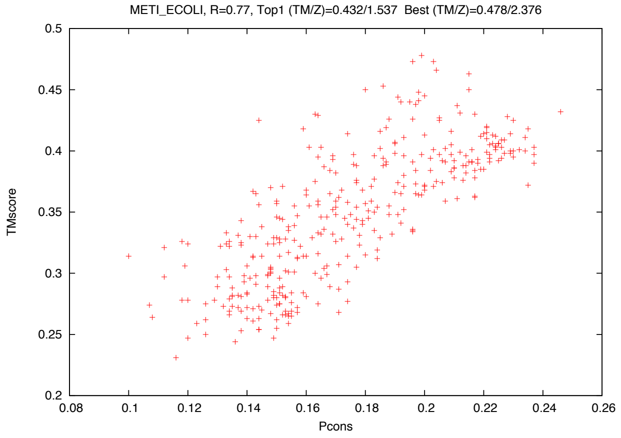
METI_ECOLI, R=0.77, Top1 (TM/Z)=0.432/1.537 Best (TM/Z)=0.478/2.376
TMscore
Pcons

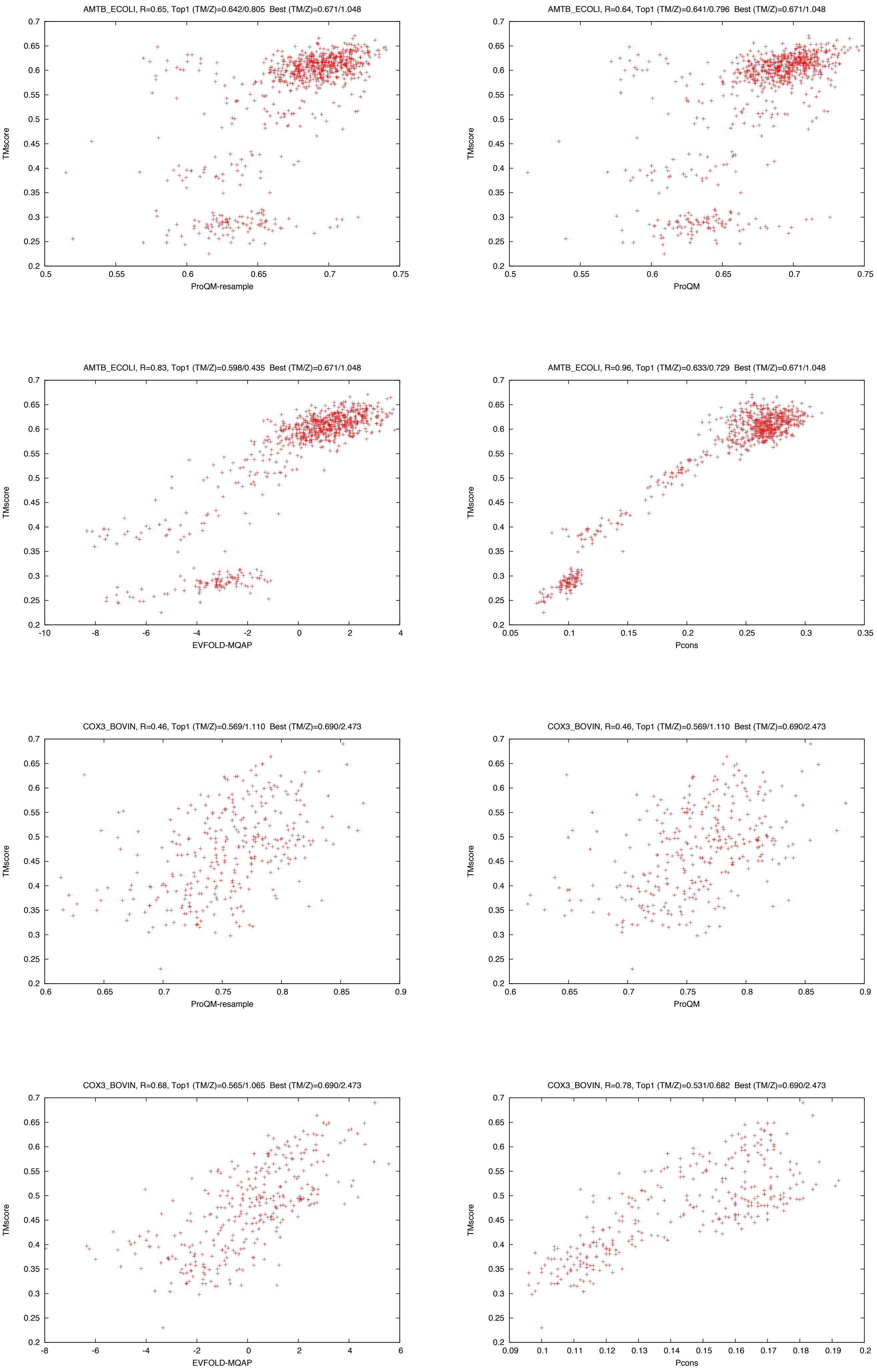
AMTB_ECOLI, R=0.65, Top1 (TM/Z)=0.642/0.805 Best (TM/Z)=0.671/1.048
TMscore
ProQM-resample
AMTB_ECOLI, R=0.64, Top1 (TM/Z)=0.641/0.796 Best (TM/Z)=0.671/1.048
TMscore
ProQM
AMTB_ECOLI, R=0.83, Top1 (TM/Z)=0.598/0.435 Best (TM/Z)=0.671/1.048
TMscore
EVFOLD-MQAP
AMTB_ECOLI, R=0.96, Top1 (TM/Z)=0.633/0.729 Best (TM/Z)=0.671/1.048
TMscore
Pcons
COX3_BOVIN, R=0.46, Top1 (TM/Z)=0.569/1.110 Best (TM/Z)=0.690/2.473
TMscore
ProQM-resample
COX3_BOVIN, R=0.46, Top1 (TM/Z)=0.569/1.110 Best (TM/Z)=0.690/2.473
TMscore
ProQM
COX3_BOVIN, R=0.68, Top1 (TM/Z)=0.565/1.065 Best (TM/Z)=0.690/2.473
TMscore
EVFOLD-MQAP
COX3_BOVIN, R=0.78, Top1 (TM/Z)=0.531/0.682 Best (TM/Z)=0.690/2.473
TMscore
Pcons

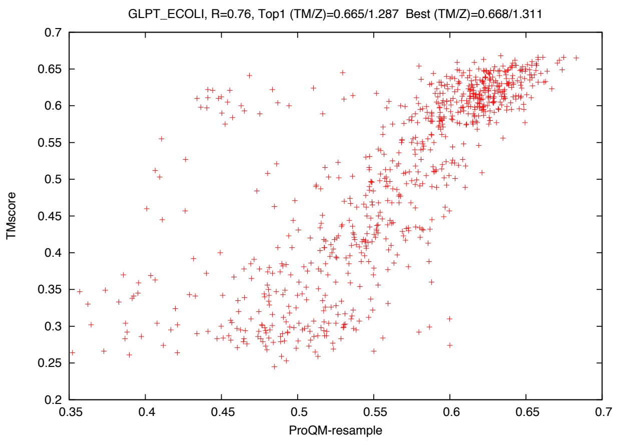
GLPT_ECOLI, R=0.76, Top1 (TM/Z)=0.665/1.287 Best (TM/Z)=0.668/1.311
TMscore
ProQM-resample

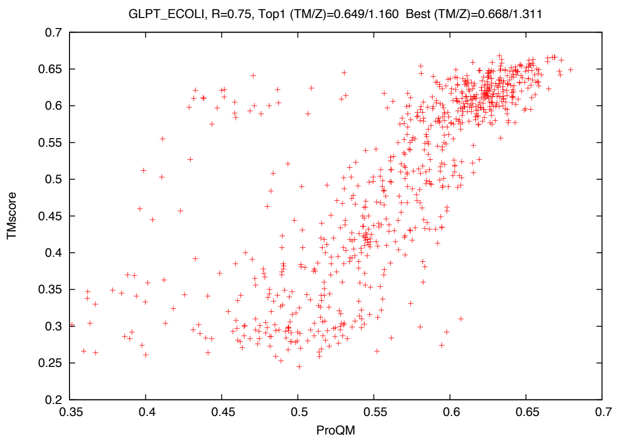
GLPT_ECOLI, R=0.75, Top1 (TM/Z)=0.649/1.160 Best (TM/Z)=0.668/1.311
TMscore
ProQM

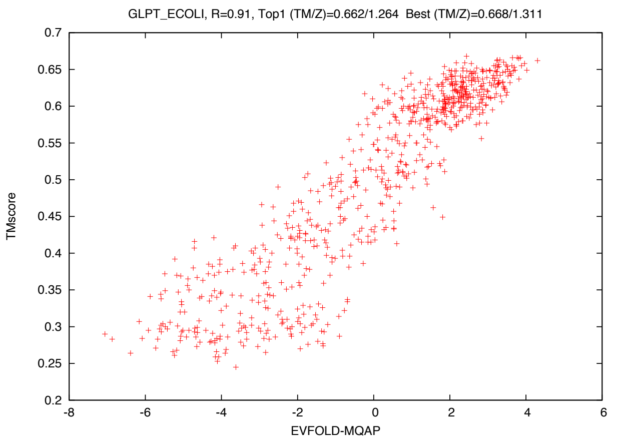
GLPT_ECOLI, R=0.91, Top1 (TM/Z)=0.662/1.264 Best (TM/Z)=0.668/1.311
TMscore
EVFOLD-MQAP

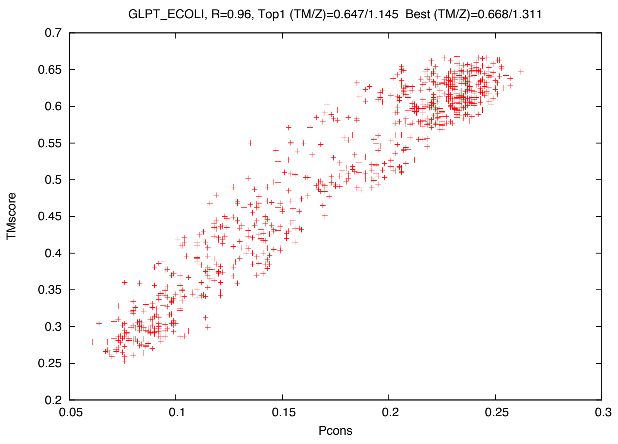
GLPT_ECOLI, R=0.96, Top1 (TM/Z)=0.647/1.145 Best (TM/Z)=0.668/1.311
TMscore
Pcons

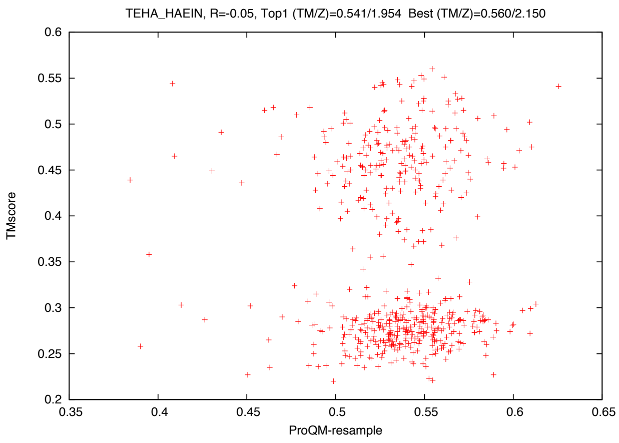
TEHA_HAEIN, R=-0.05, Top1 (TM/Z)=0.541/1.954 Best (TM/Z)=0.560/2.150
TMscore
ProQM-resample

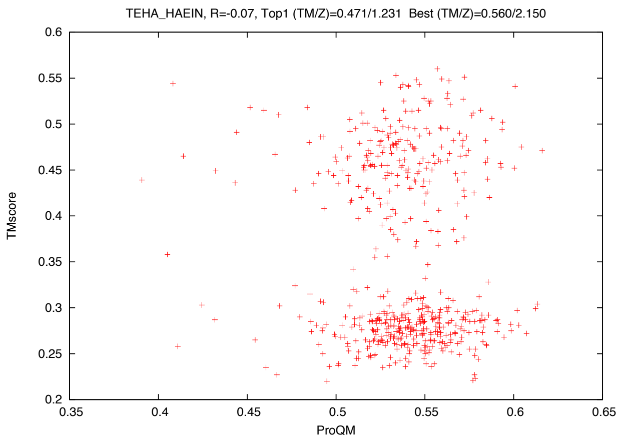
TEHA_HAEIN, R=-0.07, Top1 (TM/Z)=0.471/1.231 Best (TM/Z)=0.560/2.150
TMscore
ProQM

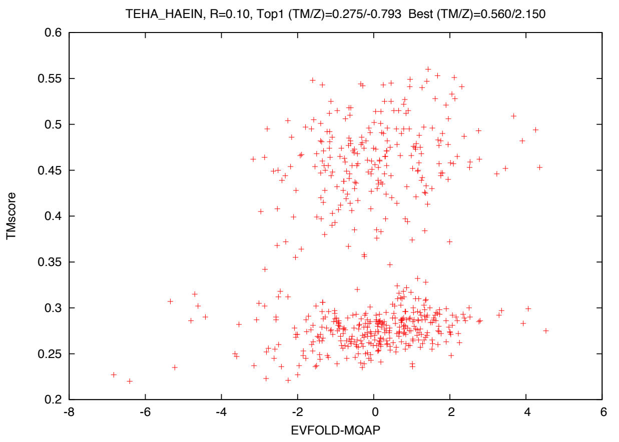
TEHA_HAEIN, R=0.10, Top1 (TM/Z)=0.275/-0.793 Best (TM/Z)=0.560/2.150
TMscore
EVFOLD-MQAP

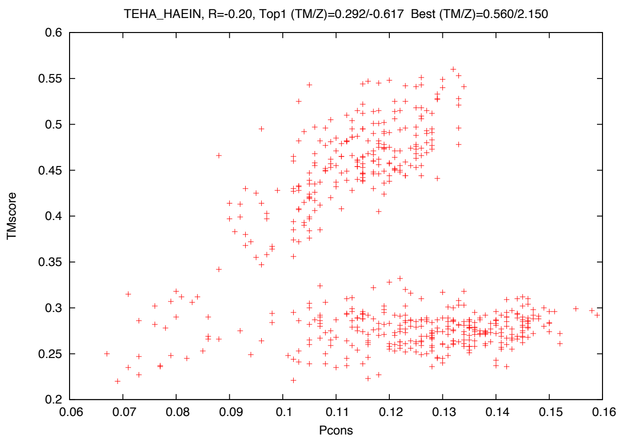
TEHA_HAEIN, R=-0.20, Top1 (TM/Z)=0.292/-0.617 Best (TM/Z)=0.560/2.150
TMscore
Pcons

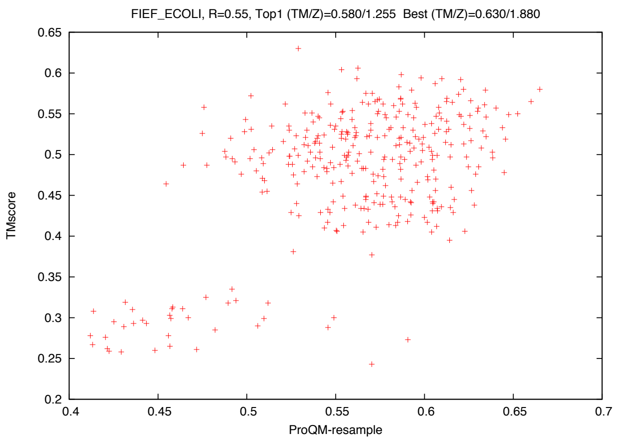
FIEF_ECOLI, R=0.55, Top1 (TM/Z)=0.580/1.255 Best (TM/Z)=0.630/1.880
TMscore
ProQM-resample

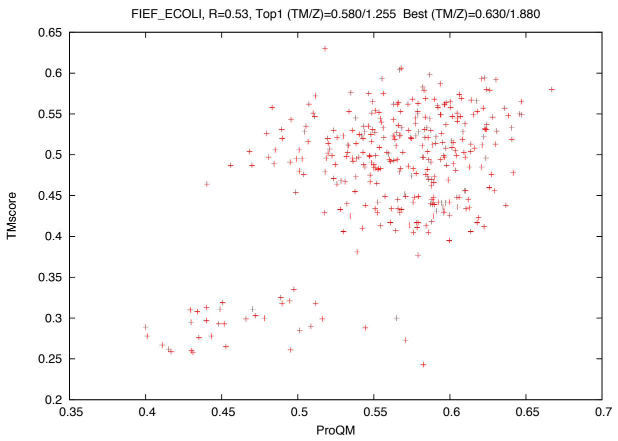
FIEF_ECOLI, R=0.53, Top1 (TM/Z)=0.580/1.255 Best (TM/Z)=0.630/1.880
TMscore
ProQM

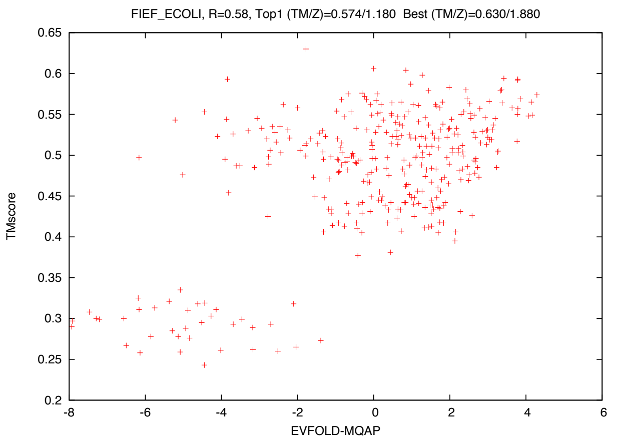
FIEF_ECOLI, R=0.58, Top1 (TM/Z)=0.574/1.180 Best (TM/Z)=0.630/1.880
TMscore
EVFOLD-MQAP

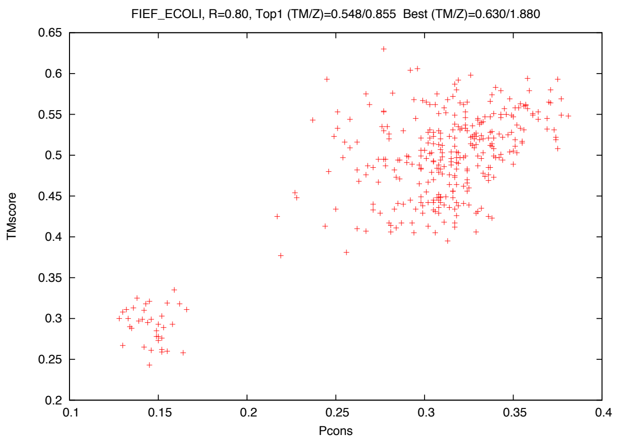
FIEF_ECOLI, R=0.80, Top1 (TM/Z)=0.548/0.855 Best (TM/Z)=0.630/1.880
TMscore
Pcons

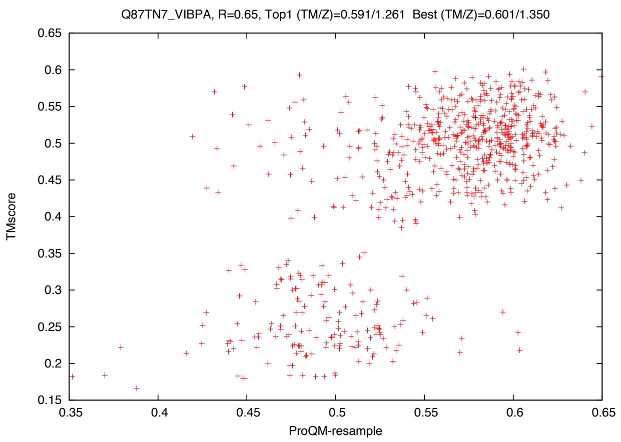
Q87TN7_VIBPA, R=0.65, Top1 (TM/Z)=0.591/1.261 Best (TM/Z)=0.601/1.350
TMscore
ProQM-resample

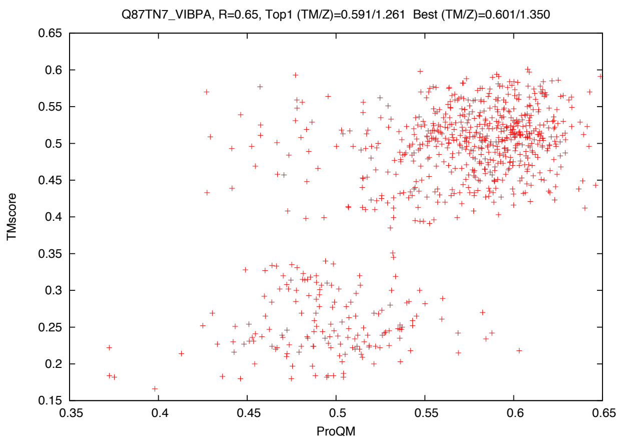
Q87TN7_VIBPA, R=0.65, Top1 (TM/Z)=0.591/1.261 Best (TM/Z)=0.601/1.350
TMscore
ProQM

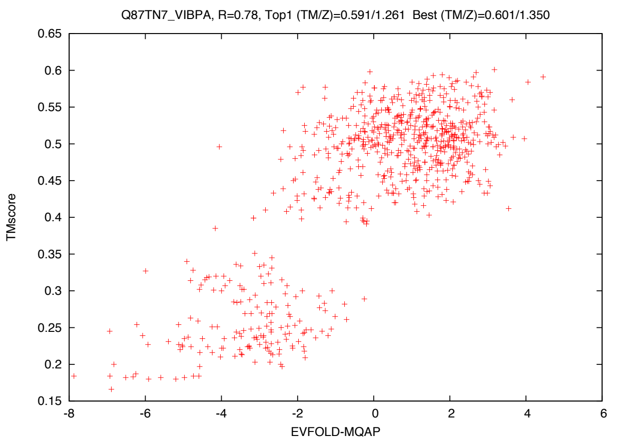
Q87TN7_VIBPA, R=0.78, Top1 (TM/Z)=0.591/1.261 Best (TM/Z)=0.601/1.350
TMscore
EVFOLD-MQAP

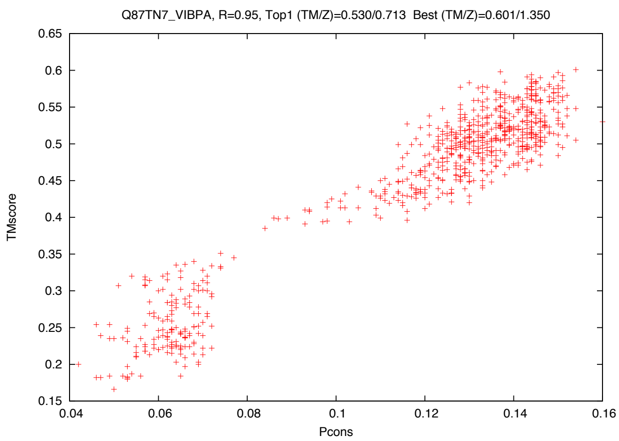
Q87TN7_VIBPA, R=0.95, Top1 (TM/Z)=0.530/0.713 Best (TM/Z)=0.601/1.350
TMscore
Pcons

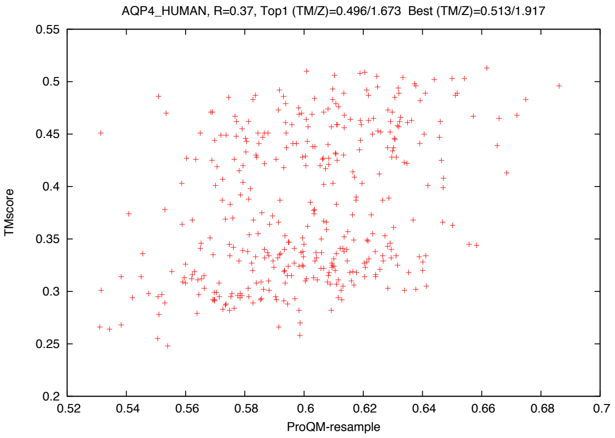
AQP4_HUMAN, R=0.37, Top1 (TM/Z)=0.496/1.673 Best (TM/Z)=0.513/1.917
TMscore
ProQM-resample

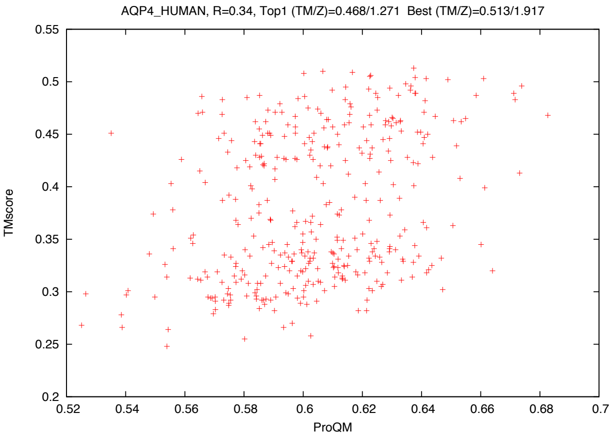
AQP4_HUMAN, R=0.34, Top1 (TM/Z)=0.468/1.271 Best (TM/Z)=0.513/1.917
TMscore
ProQM

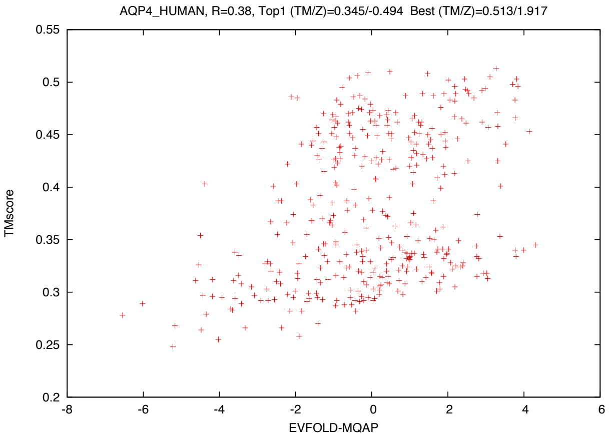
AQP4_HUMAN, R=0.38, Top1 (TM/Z)=0.345/-0.494 Best (TM/Z)=0.513/1.917
TMscore
EVFOLD-MQAP

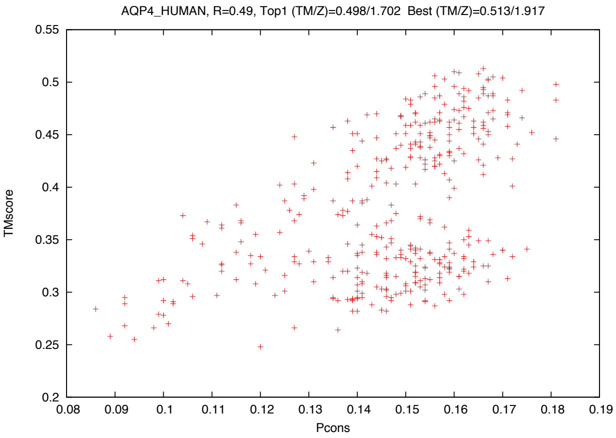
AQP4_HUMAN, R=0.49, Top1 (TM/Z)=0.498/1.702 Best (TM/Z)=0.513/1.917
TMscore
Pcons

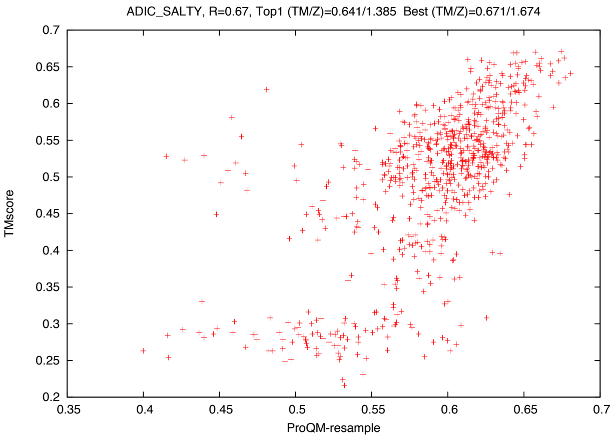
ADIC_SALTY, R=0.67, Top1 (TM/Z)=0.641/1.385 Best (TM/Z)=0.671/1.674
TMscore
ProQM-resample

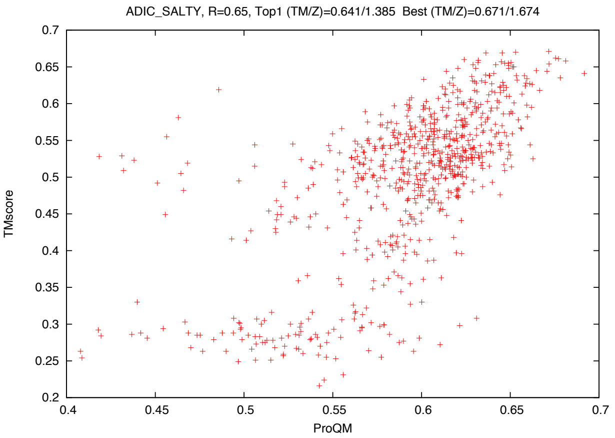
ADIC_SALTY, R=0.65, Top1 (TM/Z)=0.641/1.385 Best (TM/Z)=0.671/1.674
TMscore
ProQM

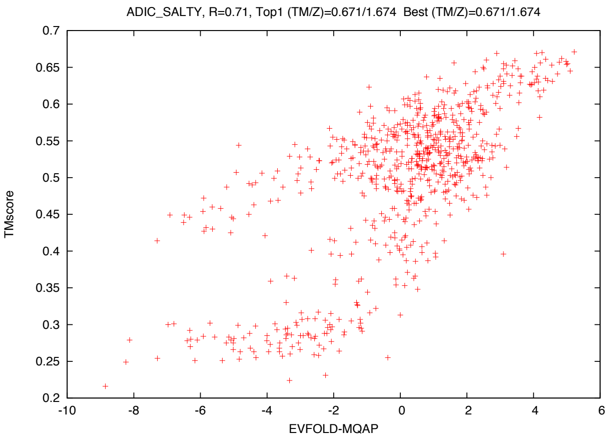
ADIC_SALTY, R=0.71, Top1 (TM/Z)=0.671/1.674 Best (TM/Z)=0.671/1.674
TMscore
EVFOLD-MQAP

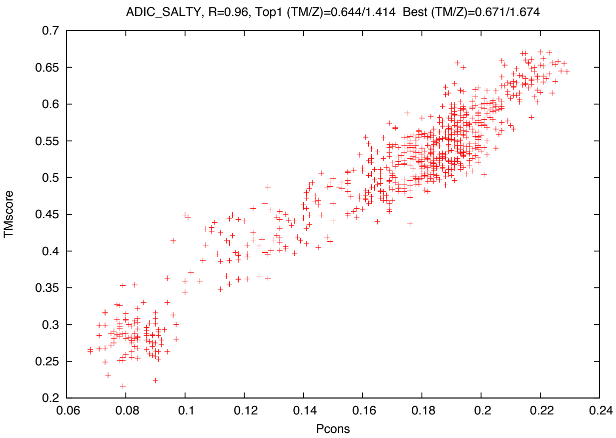
ADIC_SALTY, R=0.96, Top1 (TM/Z)=0.644/1.414 Best (TM/Z)=0.671/1.674
TMscore
Pcons

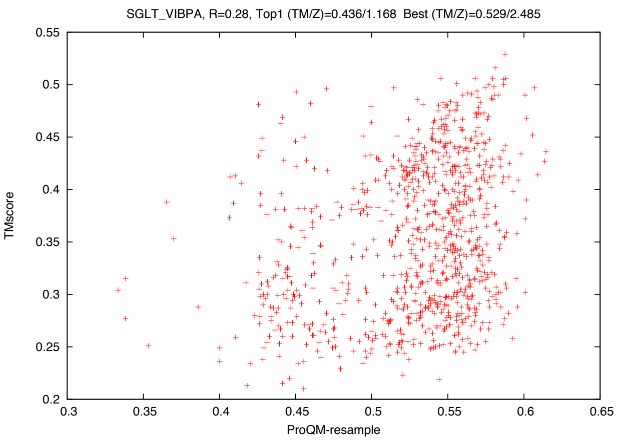
SGLT_VIBPA, R=0.28, Top1 (TM/Z)=0.436/1.168 Best (TM/Z)=0.529/2.485
TMscore
ProQM-resample

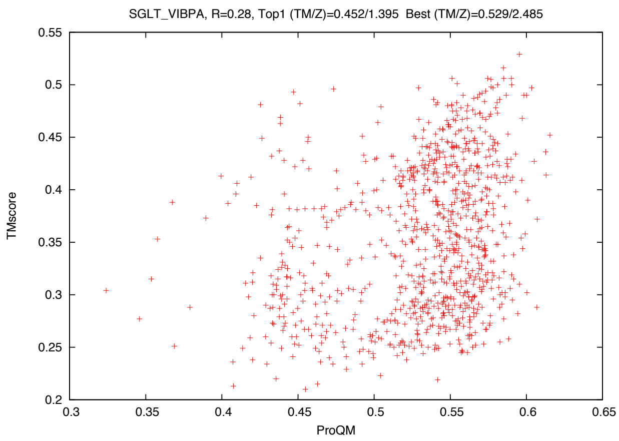
SGLT_VIBPA, R=0.28, Top1 (TM/Z)=0.452/1.395 Best (TM/Z)=0.529/2.485
TMscore
ProQM

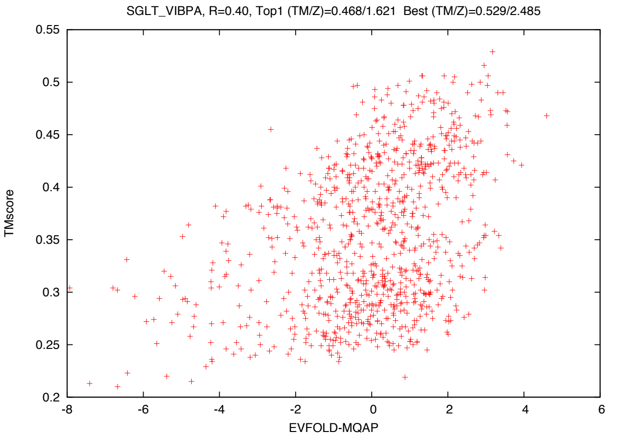
SGLT_VIBPA, R=0.40, Top1 (TM/Z)=0.468/1.621 Best (TM/Z)=0.529/2.485
TMscore
EVFOLD-MQAP

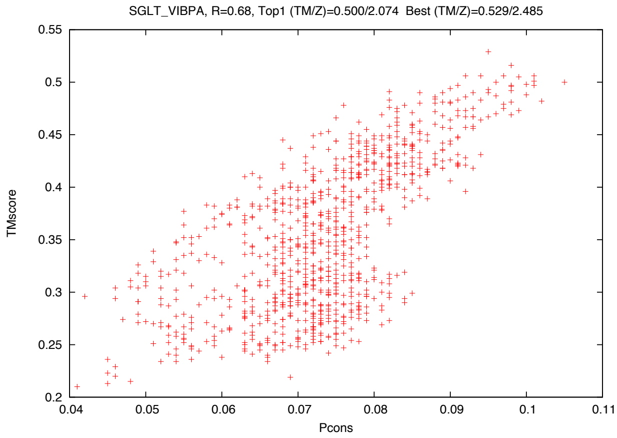
SGLT_VIBPA, R=0.68, Top1 (TM/Z)=0.500/2.074 Best (TM/Z)=0.529/2.485
TMscore
Pcons

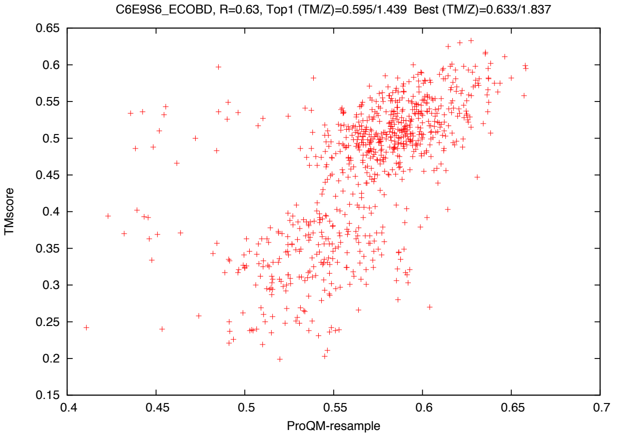
C6E9S6_ECOBD, R=0.63, Top1 (TM/Z)=0.595/1.439 Best (TM/Z)=0.633/1.837
TMscore
ProQM-resample

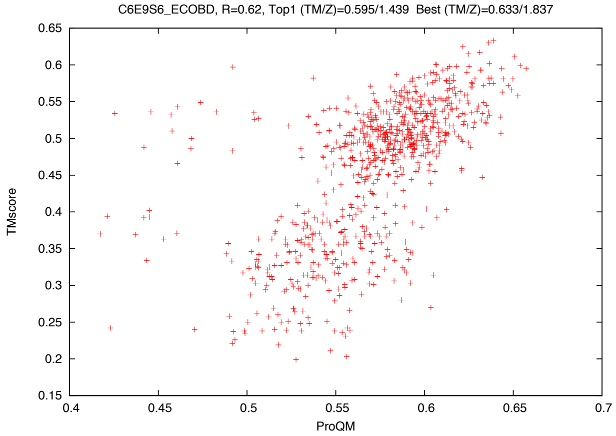
C6E9S6_ECOBD, R=0.62, Top1 (TM/Z)=0.595/1.439 Best (TM/Z)=0.633/1.837
TMscore
ProQM

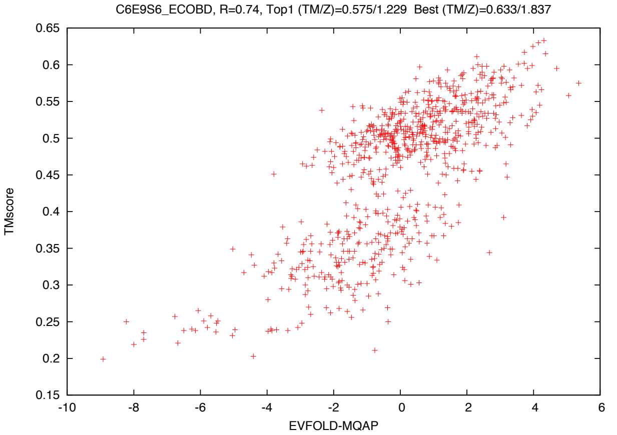
C6E9S6_ECOBD, R=0.74, Top1 (TM/Z)=0.575/1.229 Best (TM/Z)=0.633/1.837
TMscore
EVFOLD-MQAP

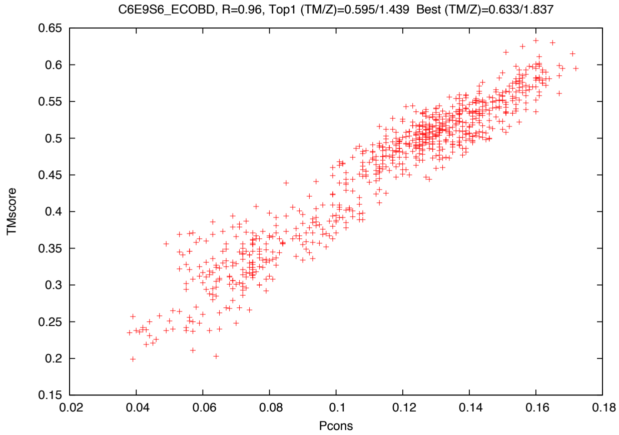
C6E9S6_ECOBD, R=0.96, Top1 (TM/Z)=0.595/1.439 Best (TM/Z)=0.633/1.837
TMscore
Pcons

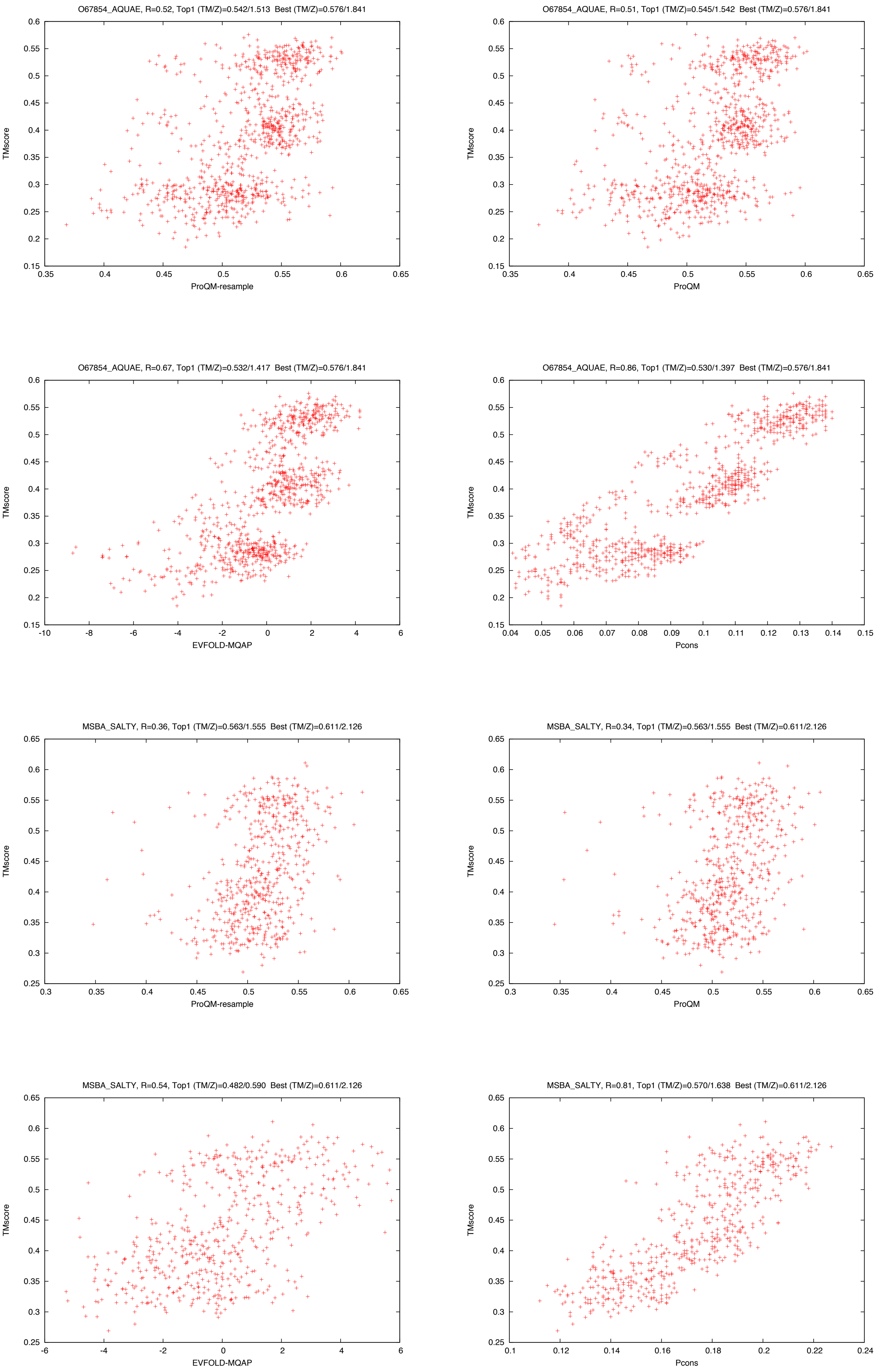
O67854_AQUAE, R=0.52, Top1 (TM/Z)=0.542/1.513 Best (TM/Z)=0.576/1.841
TMscore
ProQM-resample
O67854_AQUAE, R=0.51, Top1 (TM/Z)=0.545/1.542 Best (TM/Z)=0.576/1.841
TMscore
ProQM
O67854_AQUAE, R=0.67, Top1 (TM/Z)=0.532/1.417 Best (TM/Z)=0.576/1.841
TMscore
EVFOLD-MQAP
O67854_AQUAE, R=0.86, Top1 (TM/Z)=0.530/1.397 Best (TM/Z)=0.576/1.841
TMscore
Pcons
MSBA_SALTY, R=0.36, Top1 (TM/Z)=0.563/1.555 Best (TM/Z)=0.611/2.126
TMscore
ProQM-resample
MSBA_SALTY, R=0.34, Top1 (TM/Z)=0.563/1.555 Best (TM/Z)=0.611/2.126
TMscore
ProQM
MSBA_SALTY, R=0.54, Top1 (TM/Z)=0.482/0.590 Best (TM/Z)=0.611/2.126
TMscore
EVFOLD-MQAP
MSBA_SALTY, R=0.81, Top1 (TM/Z)=0.570/1.638 Best (TM/Z)=0.611/2.126
TMscore
Pcons

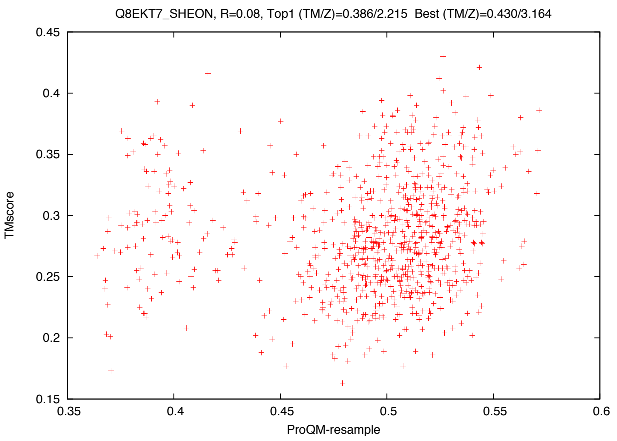
Q8EKT7_SHEON, R=0.08, Top1 (TM/Z)=0.386/2.215 Best (TM/Z)=0.430/3.164
TMscore
ProQM-resample

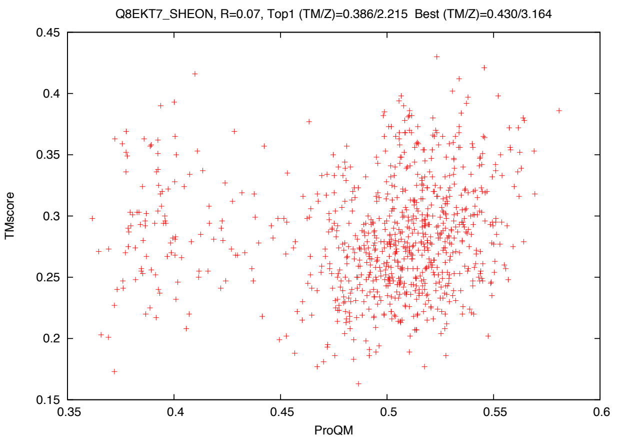
Q8EKT7_SHEON, R=0.07, Top1 (TM/Z)=0.386/2.215 Best (TM/Z)=0.430/3.164
TMscore
ProQM

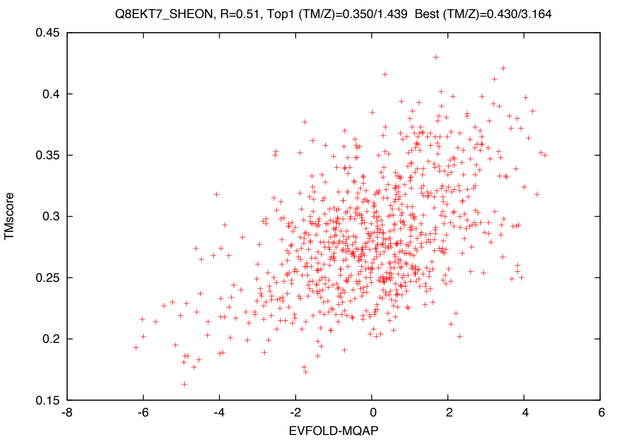
Q8EKT7_SHEON, R=0.51, Top1 (TM/Z)=0.350/1.439 Best (TM/Z)=0.430/3.164
TMscore
EVFOLD-MQAP

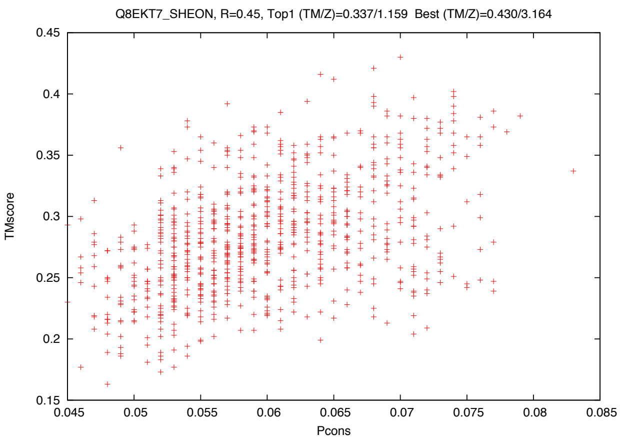
Q8EKT7_SHEON, R=0.45, Top1 (TM/Z)=0.337/1.159 Best (TM/Z)=0.430/3.164
TMscore
Pcons

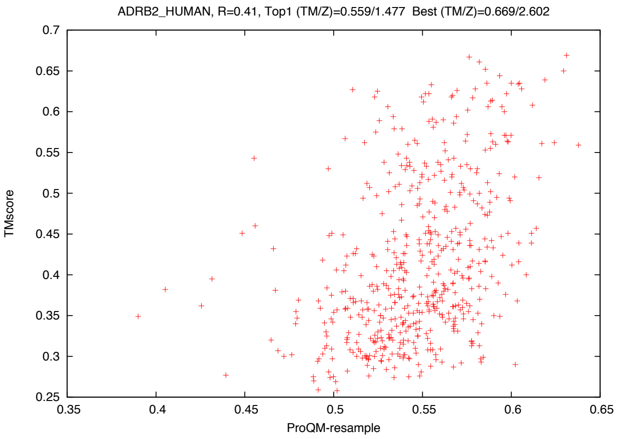
ADRB2_HUMAN, R=0.41, Top1 (TM/Z)=0.559/1.477 Best (TM/Z)=0.669/2.602
TMscore
ProQM-resample

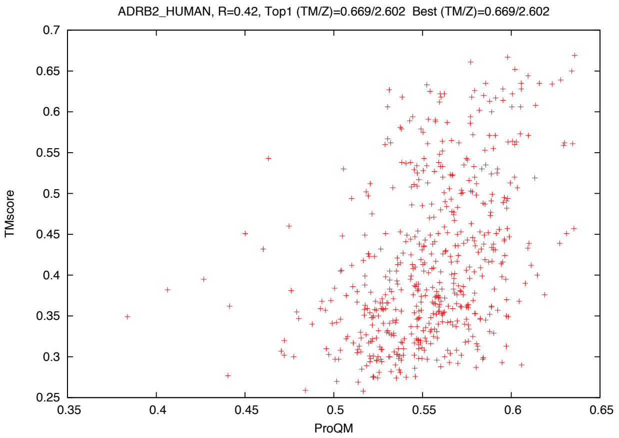
ADRB2_HUMAN, R=0.42, Top1 (TM/Z)=0.669/2.602 Best (TM/Z)=0.669/2.602
TMscore
ProQM

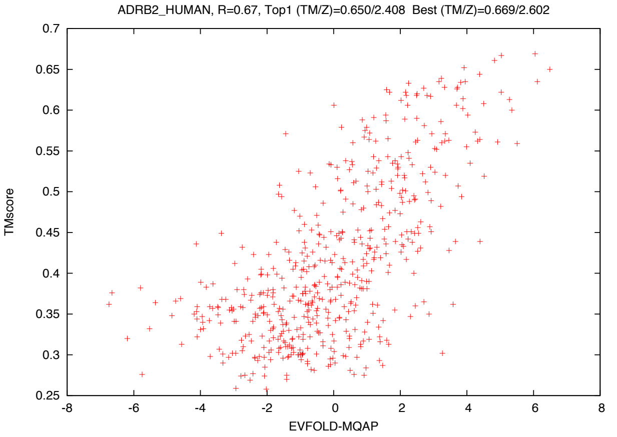
ADRB2_HUMAN, R=0.67, Top1 (TM/Z)=0.650/2.408 Best (TM/Z)=0.669/2.602
TMscore
EVFOLD-MQAP

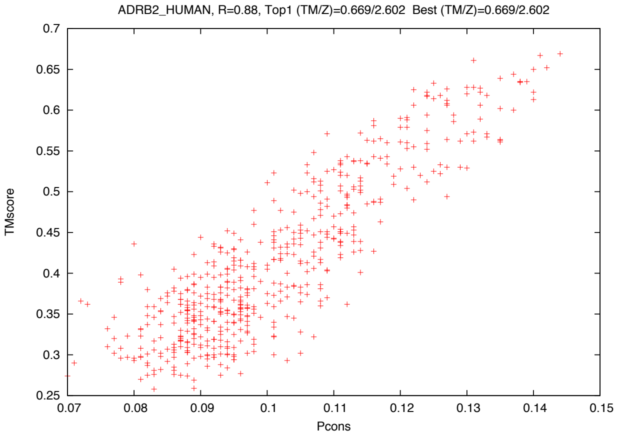
ADRB2_HUMAN, R=0.88, Top1 (TM/Z)=0.669/2.602 Best (TM/Z)=0.669/2.602
TMscore
Pcons

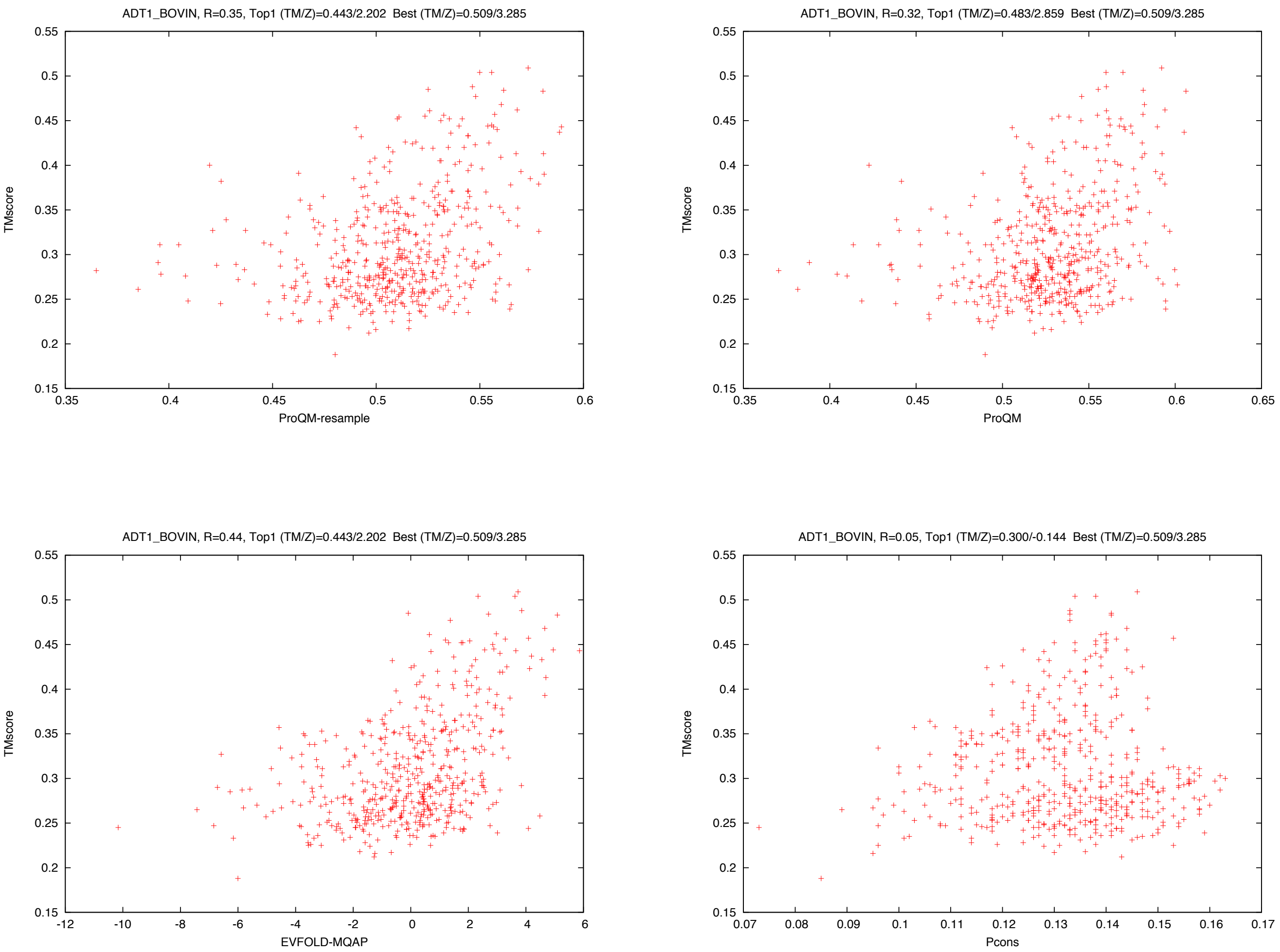
ADT1_BOVIN, R=0.35, Top1 (TM/Z)=0.443/2.202 Best (TM/Z)=0.509/3.285
TMscore
ProQM-resample
ADT1_BOVIN, R=0.32, Top1 (TM/Z)=0.483/2.859 Best (TM/Z)=0.509/3.285
TMscore
ProQM
ADT1_BOVIN, R=0.44, Top1 (TM/Z)=0.443/2.202 Best (TM/Z)=0.509/3.285
TMscore
EVFOLD-MQAP
ADT1_BOVIN, R=0.05, Top1 (TM/Z)=0.300/-0.144 Best (TM/Z)=0.509/3.285
TMscore
Pcons
